# Role of Na_2_CO_3_ as Nucleation Seeds to Accelerate the CO_2_ Uptake Kinetics of MgO-Based Sorbents

**DOI:** 10.1021/jacsau.4c00782

**Published:** 2024-11-16

**Authors:** Annelies Landuyt, Ilia Kochetygov, Charles J. McMonagle, Priyank V. Kumar, Jodie A. Yuwono, Wendy L. Queen, Paula M. Abdala, Christoph R. Müller

**Affiliations:** † Laboratory of Energy Science and Engineering, Department of Mechanical and Process Engineering, Eidgenössische Technische Hochschule (ETH) Zürich, 8092 Zürich, Switzerland; ‡ Paul Scherrer Institut, PSI Center for Energy and Environmental Sciences, Villigen, PSI CH-5232, Switzerland; § Swiss-Norwegian Beamlines (SNBL), European Synchrotron Radiation Facility (ESRF), Grenoble 38000, France; ∥ School of Chemical Engineering, The University of New South Wales (UNSW Sydney), 2052 Sydney, New South Wales, Australia; ⊥ School of Chemical Engineering, The University of Adelaide, Adelaide, SA 5005, Australia; # Institute of Chemical Sciences and Engineering (ISIC), École Polytechnique Fédérale de Lausanne (EPFL), 1051 Sion, Switzerland

**Keywords:** CO_2_ capture, MgO, carbonate, X-ray diffraction, molten salts, nucleation seed, in situ

## Abstract

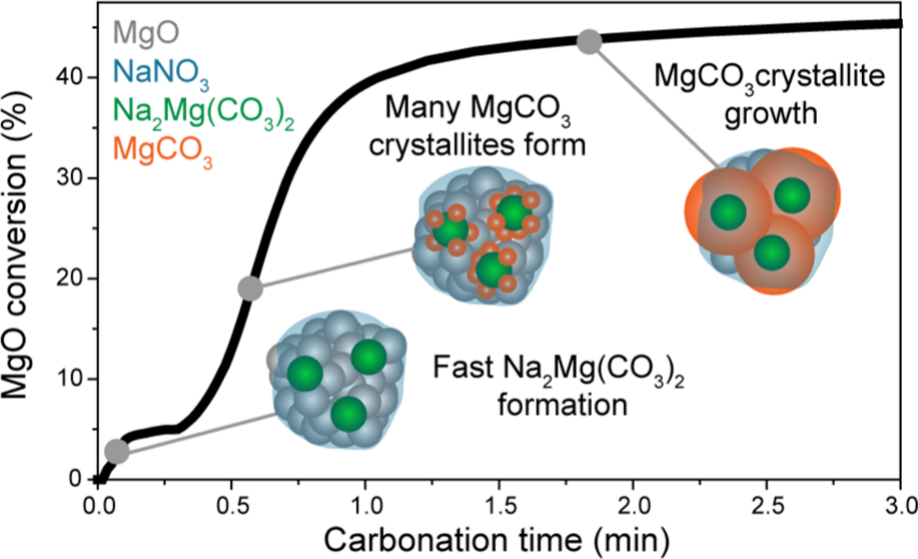

There is an urgent need for inexpensive, functional materials that can capture and release CO_2_ under industrial conditions. In this context, MgO is a highly promising, earth-abundant CO_2_ sorbent. However, despite its favorable carbonation thermodynamics and potential for high gravimetric CO_2_ uptakes, MgO-based CO_2_ sorbents feature slow carbonation kinetics, limiting their CO_2_ uptake during typical industrial contact times. The addition of molten alkali metal nitrate promoters, such as NaNO_3_, can partially mitigate the slow kinetics. Here, we investigate how the CO_2_ uptake kinetics of NaNO_3_-promoted MgO can be increased further through the addition of finely dispersed Na_2_CO_3_. The incorporation of Na_2_CO_3_ significantly increases the CO_2_ uptake rate from 1.4 to 14.6 mmol MgCO_3_ (mol MgO)^−1^ s^–1^. Using *in situ* synchrotron X-ray powder diffraction (XRD), we track the formation of MgCO_3_ and elucidate the mechanism through which Na_2_CO_3_ promotes the CO_2_ uptake of MgO. Our findings demonstrate that Na_2_CO_3_ rapidly converts within seconds into Na_2_Mg(CO_3_)_2_ during carbonation, acting subsequently as nucleation seeds for MgCO_3_ formation, in turn significantly enhancing CO_2_ uptake kinetics. Further, the presence of Na_2_Mg(CO_3_)_2_ considerably enhances the mobility of ions in the sorbent, leading to sintering of MgCO_3_. Importantly, Na_2_Mg(CO_3_)_2_ promotes MgCO_3_ formation even in the presence of molten RbNO_3_, a salt with a limited ability to dissolve [Mg^2+^···CO_3_^2–^] ion pairs, indicating that Na_2_Mg(CO_3_)_2_ lowers the critical ion pair concentration required for MgCO_3_ nucleation. Additionally, the partial dissolution of Na_2_CO_3_ in NaNO_3_ may increase the concentration of carbonate ions in the melt, further accelerating carbonation kinetics in MgO-(Na_2_CO_3_/NaNO_3_).

## Introduction

Carbon dioxide capture is a critical technology to reach the global CO_2_-emission targets and to keep global warming within a tolerable range, in particular in the hard-to-abate sectors.^1−4^ Currently, the benchmark CO_2_ capture technology is amine scrubbing.^5,6^ While amine scrubbing has been applied on the large scale for the removal of CO_2_ from natural gas, it suffers from severe limitations, including the degradation of amines into products which are toxic and/or harmful greenhouse gases as well as the relatively high energy demand for their regeneration (aqueous amine solution).^7,8^ Solid oxide sorbents such as CaO or MgO are a promising family of materials for CO_2_ capture due to their favorable thermodynamics, high theoretical CO_2_ uptake capacities, environmental benignity and earth-abundance.^9−11^ MgO-based CO_2_ sorbents have attracted particular interest because of their intermediate operation temperature (typically 250–450 °C), while CaO requires higher operating temperatures (typically 600–900 °C). The lower operating temperature of MgO can translate into reduced energy requirements (for sorbent regeneration) and may mitigate sintering effects.^12,13^ Further, MgO-based CO_2_ sorbents have shown promise for precombustion CO_2_ capture and sorption-enhanced water–gas shift reactions (SEWGS) due to their favorable, intermediate operating temperatures.^14,15^ Specifically, MgO reacts reversibly with CO_2_ forming MgCO_3_:
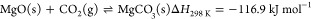
1

While the thermodynamics for this reaction are favorable, the slow kinetics for CO_2_ uptake hamper the commercial usage of MgO at present. Nonetheless, recent research has shown that the slow kinetics of CO_2_ uptake can be alleviated to some degree by promoting MgO with alkali metal nitrates (such as NaNO_3_, LiNO_3_ or mixtures of Li-, Na- and K-nitrates), yet only if the promoters are molten under operation conditions (300–450 °C).^16−18^ Although the exact mechanism through which molten alkali metal nitrates enhance the CO_2_ uptake rate of MgO is still being investigated, it is hypothesized that the molten alkali metal nitrates facilitate MgCO_3_ formation by dissolving the reactants. However, the nature and chemical environment of the dissolved species remain highly debated.^17−20^ Using ^18^O labeling and *ab initio* molecular dynamics (AIMD) calculations, a recent study revealed that when a MgO surface is exposed to a CO_2_ atmosphere, surface carbonates are readily formed. Subsequently, these surface carbonates dissolve in molten NaNO_3_ as [Mg^2+^···CO_3_^2–^] ionic pairs, which subsequently precipitate as crystalline MgCO_3_.^20^

It has been observed also that besides molten alkali metal nitrates, Na_2_CO_3_ and other alkali metal carbonates increase the rate of CO_2_ uptake of MgO.^21−24^ Since alkali metal carbonates exhibit high melting points (for instance, Na_2_CO_3_ melts at 851 °C), they are in a solid state under typical operating conditions. It has been argued that alkali metal carbonates promote the CO_2_ uptake of MgO by forming a double carbonate, a reaction with reasonable kinetics also in the absence of NaNO_3_.^25,26^ For example, Na_2_CO_3_ reacts with MgO and CO_2_ forming Na_2_Mg(CO_3_)_2_ according to
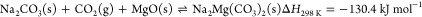
2

Generally, the promotion of MgO with alkali metal carbonates results in lower CO_2_ uptakes (0.04–0.15 g g_sorbent_^–1^) compared to its promotion with molten alkali metal nitrates (CO_2_ uptakes in the range of 0.14 to 0.6 g g_sorbent_^–1^), because their addition only leads to the formation of the respective double carbonate (e.g., Na_2_Mg(CO_3_)_2_) and not to the formation of MgCO_3_ itself.^16−18,26−28^ However, when adding a combination of alkali metal nitrates and carbonates to MgO, sorbents with both rapid CO_2_ uptake kinetics and high CO_2_ capacities are obtained.^27,29−33^ Indeed, currently the best-performing MgO-based CO_2_ sorbent is MgO promoted by a mixture of (Li, K)NO_3_ and (Na, K)_2_CO_3_ with a reported CO_2_ uptake of 0.84 g g_sorbent_^–1^ (for a carbonation time of 4 h) and a fairly stable cyclic performance, i.e., a decrease in the CO_2_ uptake by 18% over 30 cycles.^29^ Although the results of this combined promotion effort are very encouraging, we currently have only a very limited understanding of the mechanisms through which alkali metal carbonates improve the kinetics of alkali metal nitrate-promoted MgO. However, such knowledge is critical to further advance MgO-based CO_2_ sorbents and to reduce the amount of molten nitrate promoters, which may induce corrosion in the reactor vessels used.

Alkali metal carbonates can dissolve in molten nitrate salts, which has led to the hypothesis that a dissolution effect enhances the formation of double carbonates through precipitation in the melt.^27,34,35^ It was also hypothesized that the Na_2_Mg(CO_3_)_2_ formed acts as a carrier to transfer CO_2_ to MgO, thereby accelerating MgCO_3_ formation.^36^ A recent alternative mechanism suggests that Na_2_Mg(CO_3_)_2_ acts as a nucleation seed for MgCO_3_ formation owing to the structural similarity between the two carbonates structures (with *R*3̅*c* and *R*3*®H* space groups respectively).^37^ Both carbonates are described with hexagonal (or rhombohedral) lattices whose unit cells have close dimensions (MgCO_3_ has lattice parameters *a* = 4.637 Å and *c* = 15.023 Å; and Na_2_Mg(CO_3_)_2_ has lattice parameters of *a* = 4.946 Å and *c* = 16.422 Å). This structural similarity could lower the energy barrier for MgCO_3_ nucleation, resulting in faster kinetics,^38−40^ and may also explain the promoting effect of other carbonates, such as CaMg(CO_3_)_2_.^41,42^ Despite the observation of an improved CO_2_ uptake performance of such carbonate and nitrate-promoted sorbents, the underlying mechanism of the promotional effect remains elusive. Hence, gaining insight into the structural changes of sorbents under CO_2_ capture conditions is crucial for developing a detailed mechanistic understanding of the promotional effects. For example, Rekhtina et al., using *in situ* XRD and Raman spectroscopy on a MgO-NaNO_3_ sorbent, demonstrated that Na_2_Mg(CO_3_)_2_ forms through the partial decomposition of NaNO_3_ during regeneration at temperatures ≥450 °C over repeated cycles.^37^ The *in situ* formation of Na_2_Mg(CO_3_)_2_ was linked to an accelerated rate of MgCO_3_ formation, leading to the hypothesis that Na_2_Mg(CO_3_)_2_ crystallites act as seeds for MgCO_3_ nucleation and growth. However, additional mechanistic studies of the targeted addition of carbonates (such as Na_2_CO_3_) are required to fully elucidate the promotional effect of combining nitrate with carbonate promoters and to improve further the performance of MgO-based sorbents.

Hence, in this work we aim to shed light on the mechanism behind the cooperative promotional effect of Na_2_CO_3_ and NaNO_3_ on the CO_2_ uptake of MgO-based sorbents. We developed a model MgO-based CO_2_ sorbent that is promoted with both Na_2_CO_3_ and NaNO_3_, and tracked the evolution of the relevant phases (i.e., their amount, average crystallite sizes and lattice parameters) under CO_2_ capture conditions. This was achieved using time-resolved (1 s) *in situ* X-ray powder diffraction (XRD) with sufficient reciprocal space resolution to monitor the temporal evolution of the average crystallite sizes. Our experimental data revealed that Na_2_CO_3_ fully converts into Na_2_Mg(CO_3_)_2_ in just a few seconds, prior to the formation of crystalline MgCO_3_ and provides experimental evidence that Na_2_Mg(CO_3_)_2_ acts as a nucleation seed for MgCO_3_, significantly reducing the high energy barrier associated with MgCO_3_ nucleation. This, in turn, leads to a drastic increase in the nucleation and growth kinetics of MgCO_3_. Finally, the discovered promotion mechanism was validated further using MgO-RbNO_3_ as a benchmark for a poorly performing sorbent, demonstrating that the addition of Na_2_Mg(CO_3_)_2_ seeds promotes MgCO_3_ nucleation even in the presence of molten RbNO_3_, which on its own is insufficient to promote the CO_2_ uptake of MgO. These observations suggest that the role of Na_2_Mg(CO_3_)_2_ is to act as a nucleation seed, lower the critical [Mg^2+^···CO_3_^2–^] ion pair concentration in the melt required for MgCO_3_ formation and to increase the availability of [Mg^2+^···CO_3_^2–^] ions and their mobility in the sorbent via Na_2_Mg(CO_3_)_2_ dissolution in the nitrate melt.

## Results and Discussion

### Phase and Average Crystallite Size Evolution during MgO Carbonation in MgO-(Na_2_CO_3_/NaNO_3_) and MgO-NaNO_3_

**Figure 1:**
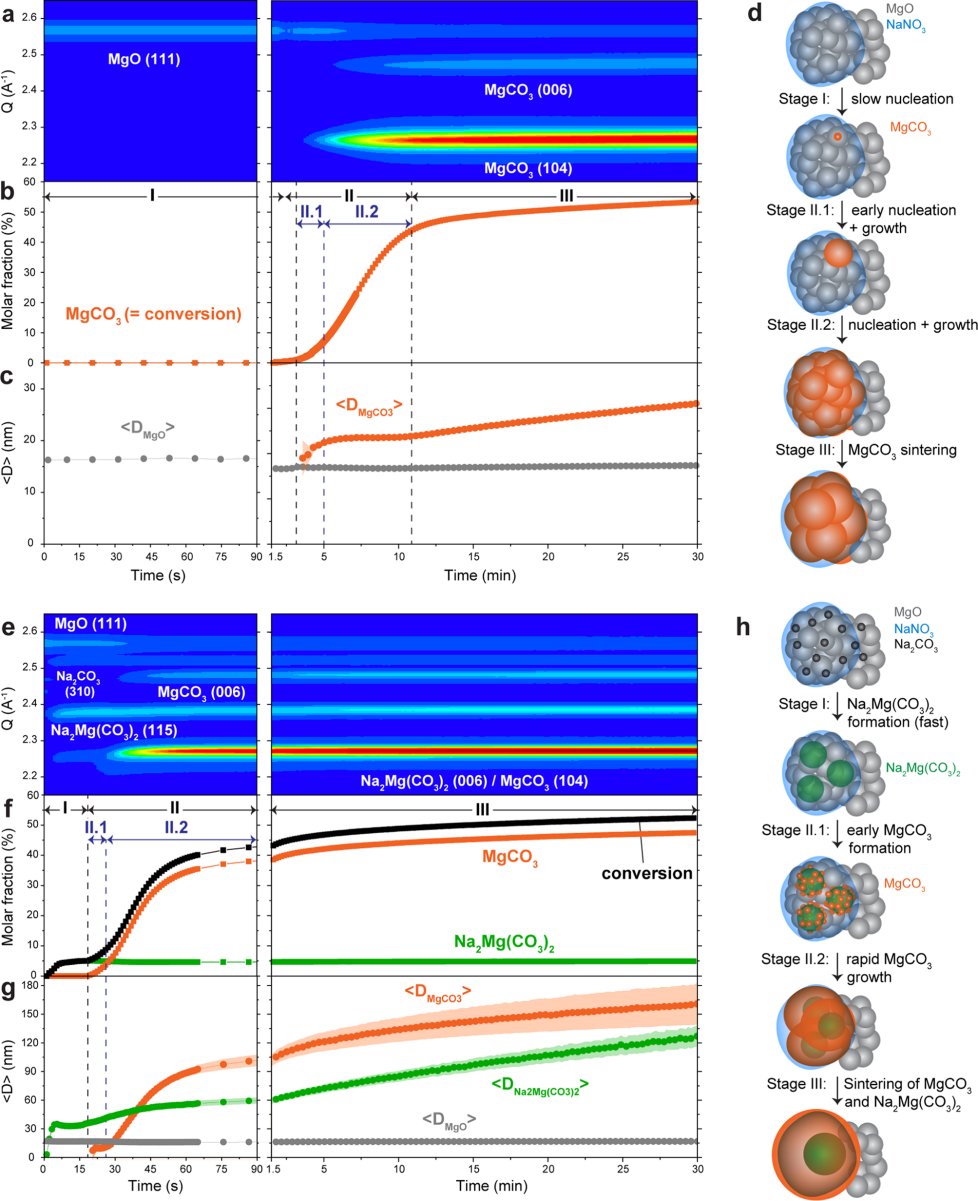
Time-resolved XRD data acquired during the carbonation of (a–d) MgO-NaNO_3_ and (e–h) MgO-(Na_2_CO_3_/NaNO_3_) at 315 °C in CO_2_. (a) 2D contour plot showing the evolution of crystalline phases during the first 90 s (left) and the remaining 30 min (right) for MgO-NaNO_3_ under carbonation conditions. (b) The molar fraction of MgCO_3_ (which is equal to MgO conversion) and (c) the average crystallite sizes of MgCO_3_ and MgO (⟨*D*_MgCO3_⟩ and ⟨*D*_MgO_⟩ respectively) as a function of time, determined from the XRD data. (d) Schematic representation of the sorbent MgO-NaNO_3_ during the different reaction stages. (e) 2D contour plot showing the evolution of the XRD peaks during the first 90 s (left) and the remaining 30 min (right) for MgO-(Na_2_CO_3_/NaNO_3_) under carbonation conditions. (f) The molar fractions of MgCO_3_ and Na_2_Mg(CO_3_)_2_ and the total MgO conversion (sum of the molar fractions of MgCO_3_ and Na_2_Mg(CO_3_)_2_) and (g) the average crystallite sizes of MgCO_3_, Na_2_Mg(CO_3_)_2_ and MgO as a function of time. (h) Schematic representation of the evolution of the sorbent MgO-(Na_2_CO_3_/NaNO_3_) during the different reaction stages. Error bars are visualized, when not visible they are smaller than the size of the symbols.

To provide mechanistic insight into how the addition of Na_2_CO_3_ affects the CO_2_ uptake of NaNO_3_-promoted MgO, we employed synchrotron-based, *in situ*, time-resolved XRD measurements (1 s time resolution) during carbonation (Figures 1 and S5). The experiments were carried out using a plug-flow capillary quartz reactor with an outer diameter of 1 mm. The high time resolution is required to resolve the rapid structural changes (phase composition, average crystallite size and lattice strain) that occur during the carbonation of the sorbents.^37,43−45^ Na_2_CO_3_/NaNO_3_-promoted MgO (referred to as MgO-(Na_2_CO_3_/NaNO_3_)) was prepared by ball milling, resulting in Na_2_CO_3_ being highly dispersed within the sorbent, as confirmed by transmission electron microscopy (TEM) and XRD analysis (Figures S1 and S2). Based on a set of experiments in which the fraction of Na_2_CO_3_ in the sorbent was varied to optimize the CO_2_ uptake and to allow for a sufficient signal intensity for XRD characterization, the optimal amount of Na_2_CO_3_ was determined as 5 mol % (Figure S4). NaNO_3_-promoted MgO (referred to as MgO-NaNO_3_) was used as a reference material and prepared via the same route, yet without the addition of Na_2_CO_3_ (Figures S2 and S3).

The *in situ* XRD data reveals that in MgO-NaNO_3_ (Figure 1a), reflections due to MgCO_3_ appear after an induction period of ca. 3 min. Rietveld analysis of the *in situ* XRD patterns (Figure S6) allows to extract the evolution of the phase composition and the average crystallite size (⟨*D*⟩) of each phase (Figure 1b,c) providing valuable insight into the prevailing crystallization process(es) during carbonation. For the following discussion it is important to note that when performing a line profile analysis of XRD data, we calculate a volume-weighted (apparent) average size of the crystallites (apparent average coherent domain size) without accounting for their size distribution.^46^ Changes in ⟨*D*⟩ during reaction could be determined with reasonable accuracy for ⟨*D*⟩ less than approximately 100 nm. The estimated standard deviation (ESD) for each refined parameter (as a function of time) is provided in the plot (Figure 1). The ESD is <1 nm for ⟨*D*⟩ less than 50 nm and <6 nm for ⟨*D*⟩ less than 100 nm. For ⟨*D*⟩ larger than 150 nm, we approach the instrumental resolution leading to higher uncertainties (ESD ca. 20 nm).

Previous works have proposed that the carbonation of NaNO_3_-promoted MgO involves the following reaction stages: surface carbonate formation, nucleation and growth of MgCO_3_ crystallites described by an Avrami–Erofeev reaction model followed by a slow carbonation stage described by a Ginstling–Brounshtein product layer diffusion process.^16,17,47,48^ We note that the phase evolution analysis (Figure 1b) performed by XRD cannot distinguish surface carbonate formation and the nucleation period, as these steps do not involve crystalline phases. Rietveld analysis of the *in situ* XRD data allowed us to divide the carbonation of NaNO_3_-promoted MgO into three stages. We define the induction period (stage I) as the period in which there is no presence of crystalline MgCO_3_. Under the conditions applied here, the induction period for MgO-NaNO_3_ lasts around 3 min and is followed by (II) MgCO_3_ nucleation and growth (3 min < *t* < 11 min) and (III) a further, slow growth of MgCO_3_, controlled by the diffusion of reactants (CO_2_ and/or carbonate ions) through the product layer (*t* > 11 min).

During carbonation of NaNO_3_-promoted MgO, the average crystallite size of MgO remains constant at ∼17 nm. In the first 2 min of stage II (labeled stage II.1), i.e., the period when the first MgCO_3_ crystallites appear and MgO reaches 6% conversion, the average crystallite size of MgCO_3_ increases rapidly to ∼23 nm and it remains constant at ∼23 nm in stage II.2 (5 min < *t* < 11 min), during which a further 39% of MgO is converted into MgCO_3_. The constant average crystallite sizes of both MgO and MgCO_3_ over time indicate that the individual MgO crystallites are fully and rapidly converted into MgCO_3_, as opposed to the simultaneous partial conversion of many MgO crystallites, which would result in a decrease in the average size of MgO. The average crystallite size of MgCO_3_ (⟨*D*_MgCO3_⟩ = 23 nm) is directly linked to the initial size of the MgO crystallites (⟨*D*_MgO_⟩ = 17 nm) when considering the expansion factor of the conversion of MgO into MgCO_3_ (⟨*D*_MgCO3_⟩ = 1.35 · ⟨*D*_MgO_⟩, see also Figure S8). The observation that the average crystallite size of MgCO_3_ does not increase with MgO conversion indicates that there is no appreciable sintering in this stage. In stage III (11 min < *t* < 30 min), the rate of MgCO_3_ formation gradually slows down, with an additional 9% of MgO being converted into MgCO_3_ (overall MgO conversion of 53%). The increase in the average crystallite size of MgCO_3_ in Stage III can primarily be attributed to a continuous liquid phase sintering process, as described previously for similar systems.^33,42,49^ Indeed, during stage III, the amount of MgCO_3_ increases by only a factor of 1.2 (from 44 to 53%), which would theoretically result in an increase in the average crystallite size of MgCO_3_ by a factor of ^3^√1.2 (∼1.06) from 23 to 24.5 nm. However, we observe an actual increase in the average MgCO_3_ crystallite size from 23 to 31 nm. A schematic representation of the structural and morphological evolution of NaNO_3_-promoted MgO during the different reaction stages during carbonation is provided in Figure 1d.

Turning now to the *in situ* XRD data of MgO-(Na_2_CO_3_/NaNO_3_) (Figure 1e–h and Figure S5 and S7), we observe a marked difference in the phase and average crystallite size evolution when compared to MgO-NaNO_3_. In MgO-(Na_2_CO_3_/NaNO_3_) crystalline MgCO_3_ is already present after 19 s, indicating a significantly shorter induction period (as compared to ca. 3 min in MgO-NaNO_3_) which is in line with thermogravimetric experiments (Figure S9). Based on the extracted phase and average crystallite size information, we can divide the carbonation of MgO-(Na_2_CO_3_/NaNO_3_) into the following three reaction stages: (I) Na_2_Mg(CO_3_)_2_ formation (*t* < 19 s), (II) MgCO_3_ nucleation and growth (19 s < *t* < 90 s) and (III) a further, slow growth of MgCO_3_, controlled by diffusion of reactants through the product layer (*t* > 90 s). In stage I, Na_2_CO_3_ and MgO rapidly react with CO_2_ to form Na_2_Mg(CO_3_)_2_, resulting in 4.5 mol % crystalline Na_2_Mg(CO_3_)_2_ after ∼19 s. This corresponds to a stoichiometric conversion, based on the Na_2_CO_3_:MgO ratio of 4.5% in the as-prepared sorbent, as determined by inductively coupled plasma optical emission spectroscopy (ICP-OES). Note that Na_2_CO_3_ was not included in the Rietveld refinement, as the peaks due to Na_2_CO_3_ were of very weak intensity at the onset of the carbonation reaction (Na_2_CO_3_ may be partially amorphous or dissolved within NaNO_3_), and these peaks disappear completely by the end of stage I. Additionally, Na_2_CO_3_ exists in two phases, which complicates further its Rietveld analysis (see Figure S10 for more details).

At t = 19 s, the stage in which MgCO_3_ rapidly nucleates and grows (stage II) begins. Initially, in stage II.1 (19 s < *t* < 25 s), small MgCO_3_ crystallites of ca. 11 nm in size form rapidly, converting 3% of MgO into MgCO_3_. Subsequently, in stage II.2 (25 s < *t* < 90 s), the MgCO_3_ crystallites rapidly grow by converting an additional 35% of MgO into MgCO_3_. The size of the MgCO_3_ crystallites at the end of stage II.2 is ∼100 nm. When comparing the dynamics of MgO conversion and MgCO_3_ crystallite size evolution in stage II.2, it is evident that the increase in the size of the MgCO_3_ crystallites is not only due to an increasing MgO conversion (i.e., increasing CO_2_ uptake) but also due to sintering. During stage II.2, the amount of MgCO_3_ increased by a factor of 13 (from 3 to 38%), which would result in an increase in the average size of the MgCO_3_ crystallites by a factor of ^3^√13 (∼2.35). However, the observed increase in the average size of the MgCO_3_ crystallites is significantly higher (by a factor of 9), i.e., a clear sign of significant sintering (Figure S11).

In stage III (*t* > 90 s) of the carbonation of MgO-(Na_2_CO_3_/NaNO_3_), the rate of MgCO_3_ formation is significantly slower, with 10% of MgO being converted into MgCO_3_ in 28.5 min (as compared to 43% MgO conversion in the first 90 s of the reaction), resulting in a total MgO conversion of 53% after 30 min of carbonation. In stage III, the average MgCO_3_ crystallite size increases from 100 to 160 nm. Again, the increase in the size of the MgCO_3_ crystallites cannot be explained solely by the increasing amount of MgCO_3_ formed but must also be due to liquid phase sintering. Compared to MgCO_3_, the sintering of Na_2_Mg(CO_3_)_2_ is even more pronounced with its average crystallite size increasing from 36 to 130 nm during stages II and III (sintering in the absence of any additional formation of Na_2_Mg(CO_3_)_2_). The pronounced sintering of Na_2_Mg(CO_3_)_2_ is likely explained by the high mobility of ions, possibly linked to Na_2_Mg(CO_3_)_2_ dissolution in molten NaNO_3_.^33,49^ Nevertheless, scanning transmission electron microscopy with energy-dispersive x-ray (STEM-EDX) maps of carbonated MgO-(Na_2_CO_3_/NaNO_3_) (Figure S13) reveal that while a fraction of Na_2_Mg(CO_3_)_2_ is in a highly agglomerated/sintered state, there is still well-distributed Na_2_Mg(CO_3_)_2_ within the sorbent. Unfortunately, it was not feasible to probe the reaction mechanism using STEM-EDX analysis due to several limitations. First, the overlapping signals of different phases (e.g., NaNO_3_ and Na_2_Mg(CO_3_)_2_ as well as MgO and MgCO_3_) made it difficult to distinguish between them. Additionally, STEM-EDX does not provide detailed structural information and certain components of the sorbent exist in different phases at room temperature compared to reaction conditions. For example, while NaNO_3_ is liquid under reaction conditions, it crystallizes into large agglomerates at room temperature.

Notably, after 30 min of carbonation the average crystallite size of MgCO_3_ is larger in MgO-(Na_2_CO_3_/NaNO_3_) compared to MgO-NaNO_3_ (160 nm versus 31 nm), although the conversion of MgO is similar in both materials. Most of the sintering in MgO-(Na_2_CO_3_/NaNO_3_) takes place during the formation of MgCO_3_ in reaction stage II, resulting in MgCO_3_ crystals with an average crystallite size of 100 nm. We propose that the dissolution of Na_2_CO_3_/Na_2_Mg(CO_3_)_2_ in NaNO_3_ facilitates the diffusion/mobility of cationic and carbonate ions (Na^+^, Mg^2+^, CO_3_^2–^) in the melt leading to an accelerated MgCO_3_ growth and liquid phase sintering.^35,50^

To assess the impact of sintering of MgCO_3_ and Na_2_Mg(CO_3_)_2_ in MgO-(Na_2_CO_3_/NaNO_3_) on its cyclic performance, we recorded the cyclic CO_2_ uptake for MgO-(Na_2_CO_3_/NaNO_3_) over 20 cycles of CO_2_ uptake and regeneration using a TGA (Figure S15). The data show that the CO_2_ uptake of MgO-(Na_2_CO_3_/NaNO_3_) decreases by 46% after 20 cycles. Nevertheless, the positive effect of the addition of Na_2_CO_3_ on the CO_2_ uptake kinetics of NaNO_3_-promoted MgO remains evident even after 20 cycles, as reflected by the faster CO_2_ uptake kinetics and overall higher CO_2_ uptake of MgO-(Na_2_CO_3_/NaNO_3_) compared to MgO-NaNO_3_.

### Evolution of MgCO_3_ Lattice Parameters and Its Implications for the Nucleation and Growth Mechanism

**Figure 2:**
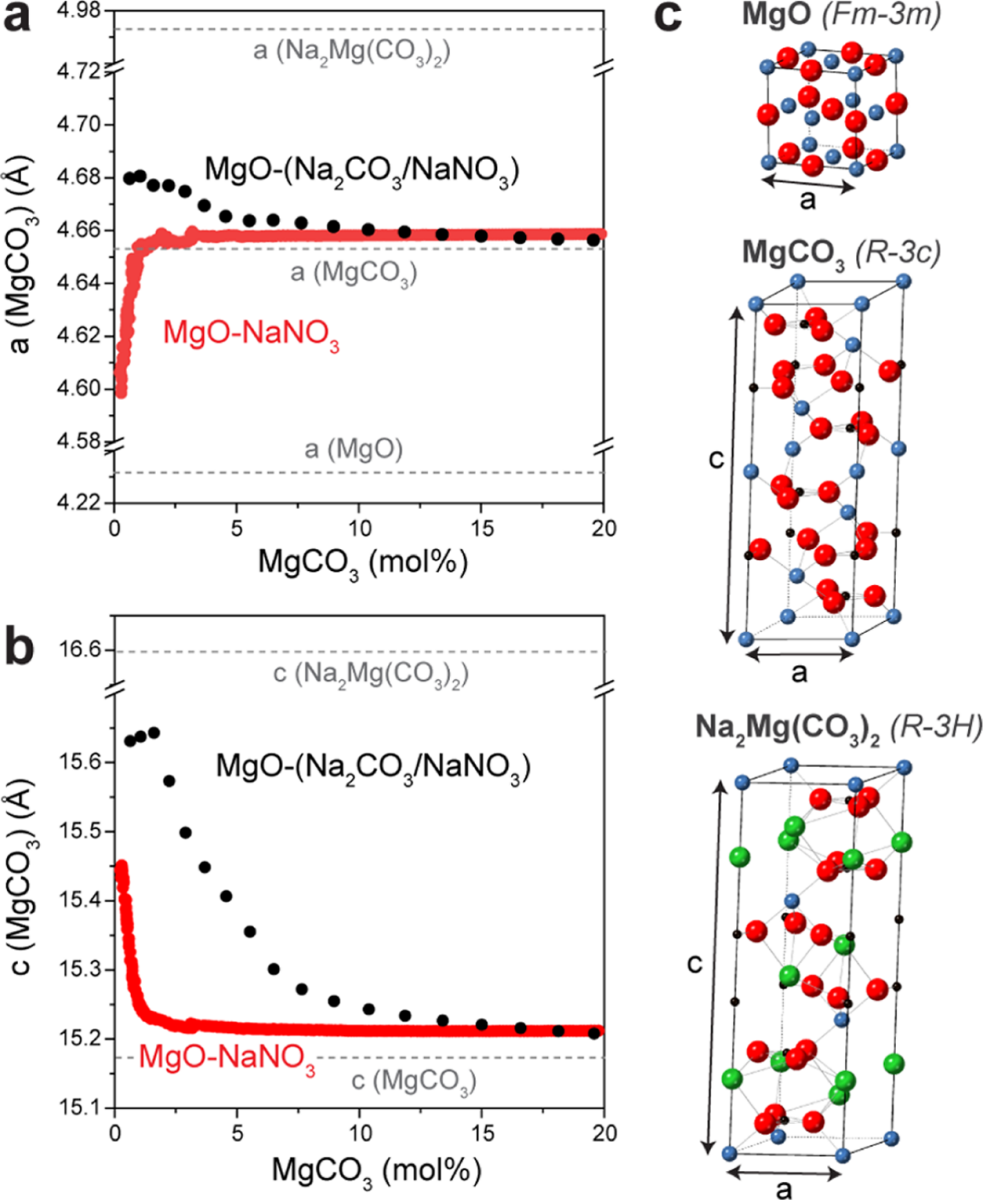
Evolution of (a) *a*-parameter and (b) *c*-parameter of MgCO_3_ during the early stages of the carbonation of MgO-NaNO_3_ (red dots) and MgO-(Na_2_CO_3_/NaNO_3_) (black dots) as a function of MgCO_3_ conversion. The respective equilibrium values (values obtained after 60 min of carbonation) of the lattice parameters of MgO, MgCO_3_ and Na_2_Mg(CO_3_)_2_ are included in the plot (gray dashed lines). The error bars are smaller than the symbols. (c) Visualization of the unit cells of MgO (*Fm*3̅*m*), MgCO_3_ (*R*3̅*c*) and Na_2_Mg(CO_3_)_2_ (*R*3̅*H*) including labeling of the respective lattice parameters.

The observation that the nucleation and growth of MgCO_3_ proceeds much faster in MgO-(Na_2_CO_3_/NaNO_3_) than in MgO-NaNO_3_ could be explained by two factors: In MgO-(Na_2_CO_3_/NaNO_3_), there is (i) a higher solubility of [Mg^2+^···CO_3_^2–^] ion pairs in molten NaNO_3_ (it has been reported that Na_2_CO_3_ has a relatively high solubility in NaNO_3_ (3.5 mol % at 315 °C and 9 mol % at 450 °C) which could lead to a higher concentration of dissolved carbonate ions in the nitrate melt, yielding in turn higher MgCO_3_ formation rates), (ii) a higher density of nucleation points for MgCO_3_ formation, or (iii) a combination of both. Our XRD measurements show that Na_2_Mg(CO_3_)_2_ formation precedes the crystallization of MgCO_3_, making it likely to act as a nucleation seed. Considering that MgCO_3_ formation is a heterogeneous nucleation process, the availability and density of nucleation points can play a key role in accelerating the MgCO_3_ formation process. Owing to the structural similarity between Na_2_Mg(CO_3_)_2_ and MgCO_3_, both crystallize in closely related rhombohedral structures (Figure 2c), Na_2_Mg(CO_3_)_2_ crystallites may act as nucleation seeds by lowering the energy barrier for MgCO_3_ nucleation, resulting in faster nucleation kinetics.^37−40^

To gain insight into the lattice strain of MgCO_3_ formed in the two sorbents, we compare the evolution of the MgCO_3_ lattice parameters obtained from Rietveld analysis of the *in situ* XRD data during the carbonation of MgO-NaNO_3_ and MgO-(Na_2_CO_3_/NaNO_3_). At the early stages of crystal growth, MgCO_3_ formed on a substrate can be strained due to a lattice mismatch with the substrate. Figure 2 plots the lattice parameters of MgCO_3_ (*a*-axis and *c*-axis) for MgO-(Na_2_CO_3_/NaNO_3_) and MgO-NaNO_3_ as a function of MgCO_3_ conversion. Note that the XRD analysis yields the average strain over all crystallites formed and not an individual crystallite lattice strain.

In the initial stages of MgCO_3_ formation, both parameters a and c differ from the values reached at high degrees of MgCO_3_ formation, confirming that MgCO_3_ is initially in a strained state that relaxes as the amount of MgCO_3_ increases. For MgO-(Na_2_CO_3_/NaNO_3_), the a-parameter of MgCO_3_ (*a* = 4.68063(6) Å) is initially larger than its final value, while for MgO-NaNO_3_, the a-parameter of MgCO_3_ (*a* = 4.59852(6) Å) is initially lower than its final value (Figure 2a). In both materials, the a-parameter relaxes with increasing conversion and the final value is ∼4.65 Å (4.65207(3) Å for MgO-(Na_2_CO_3_/NaNO_3_) and 4.65391(3) Å for MgO-NaNO_3_, see Figure S16).

Turning to the c-parameter (Figure 2b), we observe that the initial value of the c-parameter for MgO-(Na_2_CO_3_/NaNO_3_) (15.631(7) Å) is larger than for MgO-NaNO_3_ (15.433(1) Å), yet in both materials the c-parameter shows a similar trend with MgCO_3_ formation reaching ultimately an equilibrium value of *c* ∼ 15.17 Å (15.1700(3) Å for MgO MgO-(Na_2_CO_3_/NaNO_3_) and 15.1725(3) Å for MgO-NaNO_3_). As cell parameters may also depend on the size of the crystallites, we also plot the cell parameters as a function of the average crystallite size (Figure S17). Yet we cannot observe a clear correlation between the cell parameters and the average size of the MgCO_3_ crystallites.^51,52^ Instead, we hypothesize that the difference in the cell parameters of MgCO_3_ at low MgO conversions between the two sorbents arises from the different substrates onto which MgCO_3_ crystallites grow. As the initial a-parameter of MgCO_3_ in MgO-NaNO_3_ and MgO-(Na_2_CO_3_/NaNO_3_) is similar to the a-parameter of MgO and Na_2_Mg(CO_3_)_2_, respectively, we argue that in MgO-NaNO_3_, MgCO_3_ nucleates onto MgO, while in MgO-(Na_2_CO_3_/NaNO_3_), MgCO_3_ additionally nucleates onto Na_2_Mg(CO_3_)_2_. Importantly, the lattice parameter of MgO does not change during carbonation and shows similar values for both materials (*a*_(MgO)_ is 4.23101(3) Å and 4.23331(3) Å for MgO-NaNO_3_ and MgO-(Na_2_CO_3_/NaNO_3_) respectively), indicating that the observed differences in the lattice parameters of MgCO_3_ in the two sorbents studied cannot be explained by temperature or instrumental effects (Figure S18).

An analysis of the MgCO_3_ lattice parameters suggests that MgCO_3_ can nucleate on MgO in MgO-NaNO_3_ and on Na_2_Mg(CO_3_)_2_ in MgO-(Na_2_CO_3_/NaNO_3_). The lattice mismatch between the substrate and the nucleus directly influences the energy barrier for nucleation.^53,54^ For example, for the MgCO_3_–MgO interface, the lattice mismatch ε_a_ is 9.1% (ε_a_ = (*a*_MgCO_3__ – *a*_MgO_) × *a*_MgCO_3__^–1^ × 100). For MgCO_3_–Na_2_Mg(CO_3_)_2_, ε_a_ is 6.6% (ε_a_ = (*a*_MgCO_3__- *a*_Na_2_Mg(CO_3_)_2__) × *a*_MgCO_3__^–1^ × 100) and ε_c_ = 9.1 (*c*_MgCO_3__– *a*_Na_2_Mg(CO_3_)_2__) × *c*_MgCO_3__^–1^ × 100. The lower lattice mismatch for the MgCO_3_–Na_2_Mg(CO_3_)_2_ interfaces, in combination with the structural similarity between the two carbonates, could make Na_2_Mg(CO_3_)_2_ a more effective nucleation seed compared to MgO. However, further computational studies are required to rationalize in more detail the experimental findings obtained here regarding energy barriers for MgCO_3_ nucleation. Furthermore, the lattice parameter analysis does not rule out the potential contribution of small amounts of dissolved Na_2_CO_3_ in molten NaNO_3_ in increasing the kinetics of MgCO_3_ formation in MgO-(Na_2_CO_3_/NaNO_3_), e.g., by facilitating the dissolution (and potentially also increasing the concentration) of [Mg^2+^···CO_3_^2–^] ion pairs.

### Probing the Mechanism through Which Na_2_CO_3_ Promotes the Carbonation of MgO in the Presence of RbNO_3_

To understand better how Na_2_CO_3_/Na_2_Mg(CO_3_)_2_ promotes the formation of MgCO_3_, we examined the effect of the nature of the molten nitrate on the CO_2_ uptake and the dynamics of the phase evolution. We chose RbNO_3_ as it was found not to enhance the CO_2_ absorption of MgO, despite having a melting point that is very similar to that of NaNO_3_, (i.e., *T*_melting_(RbNO_3_) = 310 °C and *T*_melting_(NaNO_3_) = 308 °C).^55^ Interestingly, while MgO-RbNO_3_ shows no appreciable CO_2_ uptake, MgO-(Na_2_CO_3_/RbNO_3_) exhibits a high CO_2_ uptake at a relatively fast rate, as evidenced by thermogravimetric analysis (TGA) under carbonation conditions at 315 °C (see Figure S19).

**Figure 3:**
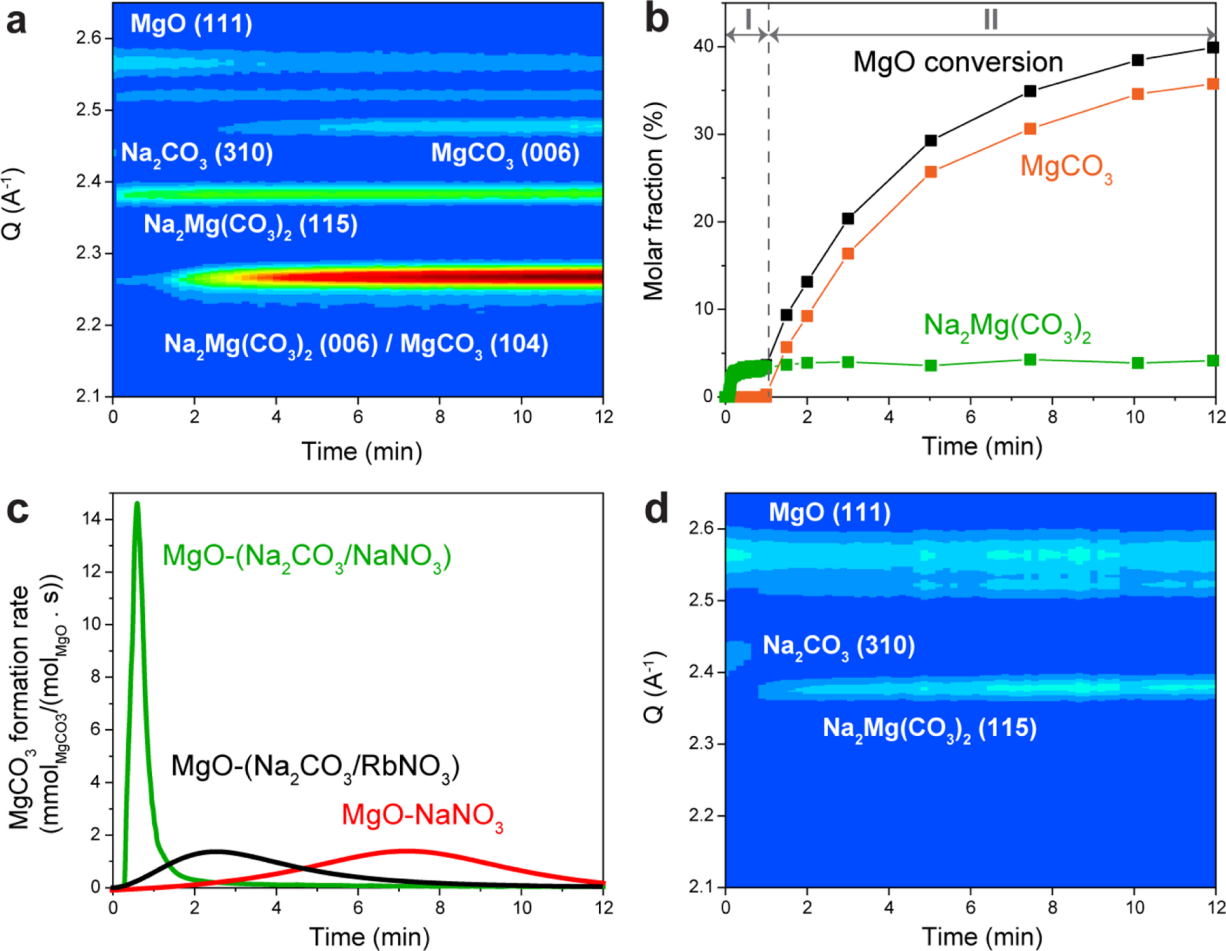
*In situ* synchrotron XRD data collected during the first 12 min of carbonation for MgO-(Na_2_CO_3_/RbNO_3_): (a) 2D contour plot of the acquired XRD patterns and (b) the molar fractions of MgCO_3_ (*x*_MgCO3_) and Na_2_Mg(CO_3_)_2_ (*x*_Na2Mg(CO3)2_) as well as the total MgO conversion (*x*_MgCO3_ + *x*_Na2Mg(CO3)2_). (c) MgCO_3_ formation rate (first derivative of the MgCO_3_ molar fraction as obtained from the *in situ* XRD data) during the first 12 min of the carbonation reaction for MgO-(Na_2_CO_3_/NaNO_3_) (green), MgO-NaNO_3_ (red) and MgO-(Na_2_CO_3_/RbNO_3_) (black). (d) 2D contour plot of the *in situ* XRD patterns (synchrotron) collected during the first 12 min of carbonation of MgO-Na_2_CO_3_.

To probe the structural evolution of MgO-(Na_2_CO_3_/RbNO_3_) under CO_2_ uptake conditions, synchrotron-based *in situ* XRD experiments were conducted (Figures 3a and S20). As the presence of Rb led to a fluorescence-induced, complex background in the XRD data and reduced the signal-to-noise ratio, the level of detail that could be extracted from the *in situ* XRD data was lower when compared to the Rb-free samples. Nonetheless, despite these limitations, we were able to extract key information on the phase evolution during the initial stages of MgCO_3_ formation. Figure 3a plots the *in situ* XRD data during the first 12 min of carbonation and Figure S21 shows the phase quantification at selected points during carbonation. Similar to MgO-(Na_2_CO_3_/NaNO_3_), we observe also for MgO-(Na_2_CO_3_/RbNO_3_) a rapid conversion of Na_2_CO_3_ into Na_2_Mg(CO_3_)_2_ during the first 20 s of carbonation (stage I), followed (after ∼1.3 min.) by the appearance of Bragg reflections due to MgCO_3_ (stage II). The amount of MgCO_3_ formed increases gradually, resulting in an overall MgO conversion of 40% after 12 min of carbonation. When compared to MgO-(Na_2_CO_3_/NaNO_3_) (Figure 1e), which reaches 40% conversion in approximately 1 min, the formation of MgCO_3_ is significantly slower in MgO-(Na_2_CO_3_/RbNO_3_). Figure 3c compares the rates of MgCO_3_ formation as a function of time for MgO-(Na_2_CO_3_/RbNO_3_), MgO-(Na_2_CO_3_/NaNO_3_) and MgO-NaNO_3_. The maximal observed rate of MgCO_3_ formation for MgO-(Na_2_CO_3_/NaNO_3_) is 14.6 mmol_MgCO_3__ mol_MgO_^–1^ s^–1^, i.e., >10 times faster than the maximal rate that is observed for MgO-(Na_2_CO_3_/RbNO_3_), viz. 1.4 mmol_MgCO_3__ mol_MgO_^–1^ s^–1^ rate. Interestingly, the maximal MgCO_3_ formation rates for MgO-(Na_2_CO_3_/RbNO_3_) and MgO-NaNO_3_ are similar, yet in MgO-(Na_2_CO_3_/RbNO_3_) the maximal rate is reached earlier, viz. after 2.5 min as compared to 7 min for MgO-NaNO_3_. This suggests that MgCO_3_ nucleation is more favorable in MgO-(Na_2_CO_3_/RbNO_3_) than in MgO-NaNO_3_.

It is important to note that the rapid and high CO_2_ uptake of MgO-(Na_2_CO_3_/RbNO_3_) cannot be explained solely by the presence of Na_2_CO_3_ or Na_2_Mg(CO_3_)_2_ as the sorbent MgO-Na_2_CO_3_ shows only a small CO_2_ uptake (Figure S19). Furthermore, the structural evolution of MgO-Na_2_CO_3_ during the first 12 min of carbonation, as obtained from synchrotron-based *in situ* XRD data (Figure 3d), reveals that the observed CO_2_ uptake is due to the formation of Na_2_Mg(CO_3_)_2_ only, as no peaks due to MgCO_3_ are observed. Even after 1 h of carbonation (Figure S22), no crystalline MgCO_3_ phase can be detected. Hence, it can be concluded that the presence of a molten salt (RbNO_3_ or NaNO_3_) is a necessary requirement for MgCO_3_ formation. It is also noted that the formation of Na_2_Mg(CO_3_)_2_ is significantly slower in the absence of a molten salt.

The dissolution of [Mg^2+^···CO_3_^2–^] ion pairs in molten alkali metal nitrates has been established as a key step in the formation of crystalline MgCO_3_ for NaNO_3_-promoted MgO-based CO_2_ sorbents.^20^ Thus, an explanation for the observation that RbNO_3_ does not promote the CO_2_ uptake of MgO on its own, while it does in the presence of Na_2_Mg(CO_3_)_2_, could be related to a low solubility of [Mg^2+^···CO_3_^2^] ion pairs in RbNO_3_ as compared to NaNO_3_. To verify this hypothesis, we performed AIMD calculations. Previous AIMD calculations reported by Landuyt et al. have determined that the dissolution energy of [Mg^2+^···CO_3_^2–^] ion pairs in NaNO_3_ (*E*_MgCO3/NaNO3_) is 1.1 eV.^20^ Here, using an identical approach, we determined a dissolution energy of [Mg^2+^···CO_3_^2–^] ion pairs in RbNO_3_ (*E*_MgCO3/RbNO3_) of 2.61 eV (Figure S23), which is approximately 2.5 times higher than *E*_MgCO3/NaNO3_. The higher energy barrier for [Mg^2+^···CO_3_^2–^] ion pair dissolution in MgO-(Na_2_CO_3_/RbNO_3_) results in a lower [Mg^2+^···CO_3_^2–^] ion pair concentration, preventing any XRD-detectable nucleation of MgCO_3_.

### Carbonation Mechanisms

**Figure 4:**
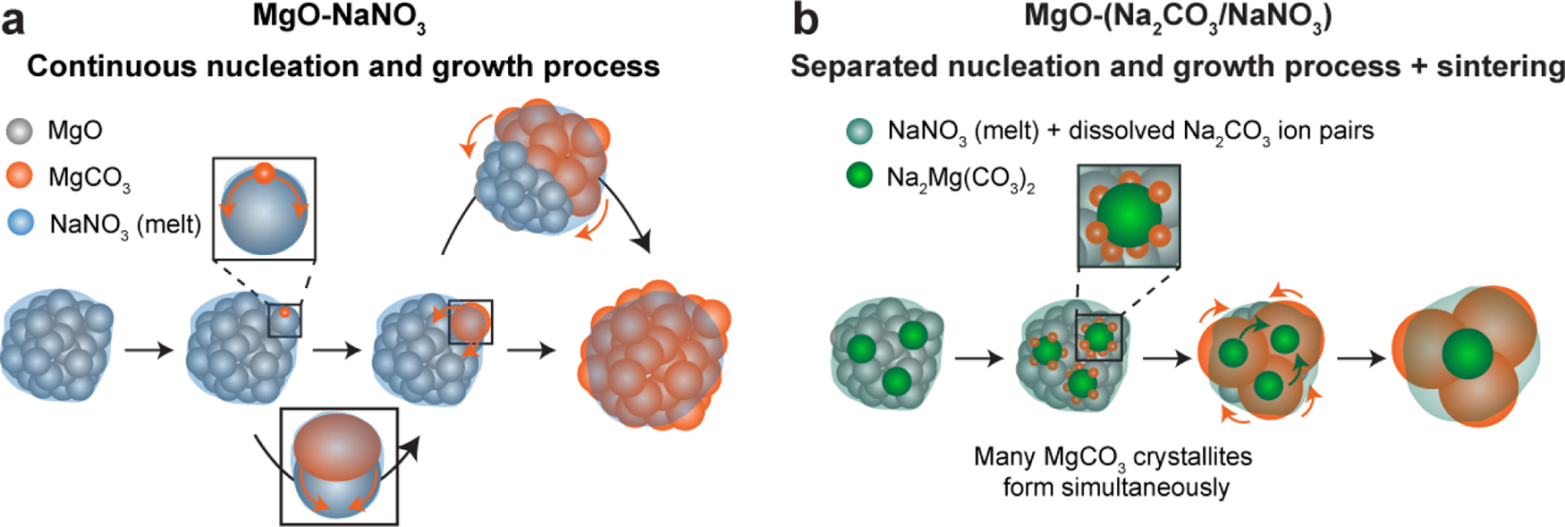
Schematic representation of the proposed MgCO_3_ formation mechanisms for (a) NaNO_3_-promoted MgO and (b) (Na_2_CO_3_/NaNO_3_)-promoted MgO.

The results presented above provide key insights into the mechanisms of MgCO_3_ formation in MgO-NaNO_3_ and MgO-(Na_2_CO_3_/NaNO_3_), suggesting fundamental differences between the mechanisms at play in these two sorbents. In MgO-NaNO_3_, the rate of MgCO_3_ formation is controlled by the nucleation of MgCO_3_ as evidenced from the relatively long induction period in combination with the observation of a continuous conversion of individual MgO crystallites into MgCO_3_. Initially, only a few MgCO_3_ crystallites are formed, that grow into larger crystallites, while simultaneously promoting MgCO_3_ nucleation in neighboring MgO crystallites. This leads to a continuous nucleation and growth process (or autocatalytic crystallization process), as illustrated in Figure 4.^41,56^ Such an autocatalytic growth process could result from a facilitated nucleation process on sites in proximity to the newly formed MgCO_3_ crystallites.

In MgO-(Na_2_CO_3_/NaNO_3_), the induction period of MgCO_3_ formation is significantly shorter (19 s as compared to 3 min in MgO-NaNO_3_) and the maximal rate of MgCO_3_ formation is approximately 10 times higher than for MgO-NaNO_3_. Furthermore, *in situ* XRD shows that the formation of Na_2_Mg(CO_3_)_2_ crystallites precedes the formation of MgCO_3_. Therefore, the Na_2_Mg(CO_3_)_2_ crystallites act as nucleation seeds for MgCO_3_ formation, greatly facilitating MgCO_3_ nucleation (most likely by lowering the energy barrier for MgCO_3_ nucleation), as illustrated in Figure 4b.

However, the presence of Na_2_CO_3_ or Na_2_Mg(CO_3_)_2_ only is not sufficient to effectively promote MgCO_3_ formation as shown by the poor CO_2_ uptake performance of MgO-Na_2_CO_3_. Instead, a molten phase is required to dissolve the reactants, most likely in the form of [Mg^2+^···CO_3_^2–^] ion pairs, that subsequently crystallize as MgCO_3_. Notably, the addition of Na_2_CO_3_ which rapidly converts into Na_2_Mg(CO_3_)_2_, allows for the formation of MgCO_3_ when using a molten nitrate (RbNO_3_) that on its own does not lead to an appreciable MgCO_3_ formation due to a relatively high energy barrier for the dissolution of [Mg^2+^···CO_3_^2–^] ion pairs in RbNO_3_. We hypothesize that the presence of nucleation seeds allows for the nucleation of MgCO_3_ at significantly lower concentrations of [Mg^2+^···CO_3_^2–^] ion pairs.^57^ The enhanced crystallization rate of MgCO_3_ could simultaneously be a result of a higher initial concentration of carbonate ions in the molten nitrates (NaNO_3_ or RbNO_3_) due to the presence of dissolved Na_2_CO_3_. The slower rate of MgCO_3_ formation in MgO-(Na_2_CO_3_/RbNO_3_) compared to MgO-(Na_2_CO_3_/NaNO_3_) indicates that the rate and concentration of ion dissolution in the melt, which is strongly affected by the nature of the salt (confirmed by AIMD) is a critical parameter for the rate of MgCO_3_ formation. Overall, these studies suggest that the combined effect of Na_2_Mg(CO_3_)_2_ seeding and a high dissolution of ions in the melt explain the promotional effect of adding Na_2_CO_3_. As reported previously, other carbonates (such as CaCO_3_, BaCO_3_ and K_2_CO_3_) also promote the formation of MgCO_3_.^32,42,58^ Future studies should assess whether also such (double) carbonates have a structural similarity with MgCO_3_ and whether they can act as nucleation seeds.

## Conclusions

In this work, we elucidated the mechanism through which the addition of Na_2_CO_3_ enhances and accelerates the CO_2_ uptake of NaNO_3_-promoted MgO. Utilizing *in situ* synchrotron-based XRD with a high time resolution, we observed that Na_2_CO_3_ converts rapidly into Na_2_Mg(CO_3_)_2_, i.e., within seconds during carbonation and precedes the formation of MgCO_3_. Na_2_Mg(CO_3_)_2_ crystallites act as nucleation seeds, accelerating the nucleation of MgCO_3_ and significantly enhancing the CO_2_ uptake kinetics. Specifically, the maximal CO_2_ uptake rate increases from 1.4 to 14.6 mmol MgCO_3_ (mol MgO)^−1^ s^–1^ when Na_2_CO_3_ is added to NaNO_3_-promoted MgO. Additionally, the presence of a high concentration of carbonate ions in the melt, due to a partial dissolution of Na_2_CO_3_ in NaNO_3_, further accelerates the carbonation kinetics in MgO-(Na_2_CO_3_/NaNO_3_). We also observed that the addition of Na_2_Mg(CO_3_)_2_ as nucleation seeds promotes MgCO_3_ formation even in the presence of a molten salt (RbNO_3_) that poorly dissolves [Mg^2+^···CO_3_^2–^] ion pairs, likely by lowering the critical ion pair concentration required for MgCO_3_ nucleation. These findings open new possibilities for the development of more effective MgO-based CO_2_-sorbents by using promoter mixtures that contain nucleation seeds or lead to the rapid, *in situ* formation of nucleation seeds.

## Experimental Section

### Materials

Magnesium nitride (Mg_3_N_2_, 99.5%), sodium nitrate (NaNO_3_, 99.5%) and rubidium nitrate (RbNO_3_, 99.7%) were purchased from Sigma-Aldrich. Sodium carbonate (Na_2_CO_3_, 99.5%) and magnesium nitrate hexahydrate (Mg(NO_3_)_2_·6H_2_O, ≥99%) were purchased from Acros Organics.

### Preparation of the Sorbents

MgO was prepared by calcining high surface area Mg(OH)_2_ (synthesized through a stoichiometric reaction of Mg_3_N_2_ with H_2_O at room temperature) for 4 h at 500 °C in air. For the preparation of Na_2_CO_3_-promoted MgO, the following amounts of precursor were used: 0.2 g Na_2_CO_3_, 1.5 g MgO (Na_2_CO_3_:MgO molar ratio of 0.05) and 10 mL isopropanol. The precursors were mixed and ball-milled with a Pulverisette 7 planetary micro mill at 100 rpm for 20 h (60 cycles of 10 min, followed by a 10 min break) using 1 mm ZrO_2_ balls in a Si_3_N_4_ cylinder. After ball milling, the sample was collected in a crystallization dish and dried at 50 °C and 1 mbar for 2 h. For unpromoted MgO, a similar milling procedure was used but without the addition of Na_2_CO_3_. To obtain the alkali metal nitrate-promoted sorbents, the milled materials were mixed with 10 mol % NaNO_3_ or RbNO_3_ by grinding for 20 min with a mortar and pestle.

### Evaluation of Sorbents

CO_2_ capture experiments were carried out in a TGA DSC3 + instrument. In a typical experiment, 15 mg of sample was loaded into an alumina crucible. The sample was first pretreated at 450 °C for 30 min in N_2_, followed by carbonation at 315 °C for 1 h in CO_2_ and cooldown to room temperature in CO_2_. The flow rate of CO_2_ or N_2_ was set to 80 mL min^–1^ and the heating and cooling rates were set to 50 °C min^–1^.

### Characterization

*Ex situ* XRD patterns were collected using a PANalytical Empyrean X-ray powder diffractometer equipped with a Bragg–Brentano HD mirror and operated at 45 kV and 40 mA using Cu Kα radiation. The scans were collected in the range of 20–70° using a step size of 0.033° and a time per step of 2.4 s. (S)TEM images and STEM-EDX maps were acquired on a Talos F200X microscope at 200 kV equipped with 4 Super-X EDS detectors. ICP-OES analysis was performed on an Agilent 5100 VDV instrument to determine the Na:Mg ratio in the as-prepared Na_2_CO_3_-promoted sorbent.

### *In Situ* Synchrotron XRD Experiments

*In situ* XRD experiments were performed at the BM01 of the European Synchrotron Radiation Facility (ESRF, Grenoble, France).^59^ The monochromatic X-ray beam with an energy of 17.8 keV (λ = 0.69668 Å) was focused to a size of 0.5 × 0.5 mm^2^ and the data was acquired using a PILATUS 2 M 24-0111 detector. XRD patterns were collected at a sample-to-detector distance of ca. 34 cm (Qmax = 8 Å^–1^, Q is the modulus of the scattering vector; Q = 4π/λ·sin α). The *in situ* experiments were carried out using a quartz capillary cell (1 mm diameter and a wall thickness of 0.01 mm) in which the material (ca. 2 mg) was fixed using quartz wool (see Figure S24 for an image of the setup). The temperature of the sample was controlled using a gas blower, which was calibrated using a thermocouple placed inside an empty quartz capillary. The sample-to-blower distance was fixed in the setup allowing for high reproducibility of the temperature and thus the determined cell parameters. Data acquisition was performed during pretreatment, carbonation and cooling down. The samples were first pretreated at 450 °C for 30 min under a flow of He (7.1 mL min^–1^) to remove any adsorbates (e.g., CO_2_ or hydroxyl groups) from the MgO surface. Subsequently, the samples were cooled down to the carbonation temperature of 315 °C. At 315 °C, the gas flow was switched to a flow of CO_2_ (13.5 mL min^–1^) for a duration of 60 min unless stated otherwise. During pretreatment and cooling down, data was acquired every 3 min (acquisition time: 1 s), during carbonation, data were acquired every 1 s. A LaB_6_ NIST (0.3 mm) diffraction standard was used for calibration of the instrument. Data normalization and azimuthal integration was performed using the SNBL software Bubble.^59^

#### Rietveld Refinement of the In Situ XRD Data during Carbonation

Parametrical Rietveld refinement of the *in situ* XRD data was performed using the software TOPAS 6.0 (Bruker AXS).^60^ The weight percentages, cell parameters and average crystallite sizes of each phase were determined as a function of time. In general, every 10th scan was used for refinement to reduce the amount of data to be analyzed. For the material (Na_2_CO_3_/NaNO_3_)-MgO, every scan was refined during the first 60 s of the carbonation reaction, to follow the rapid dynamics. Subsequently, every 10th scan was used. For the refinement, the crystal structures of MgCO_3_ (ICSD-40119), MgO (ICSD-101007) and Na_2_Mg(CO_3_)_2_ (ICSD-100482) from the Inorganic Crystal Structure Database (ICSD) were used. The determination of the MgO conversion was based on Rietveld refinement. Here, first the weight percentages of the phases, obtained through Rietveld refinement, were converted into molar percentages. Subsequently, the MgO conversion was calculated as (*n*_MgO_(*t* = 0) – *n*_MgO_(*t* = *x*)) *×n*_MgO_(*t* = 0)^−1^ × 100%, where *n*_MgO_(*t* = 0) is the molar percentage of MgO at time = 0 and *n*_MgO_(*t* = *x*) is the molar percentage of MgO after *x* seconds of carbonation. It should be noted that this calculation is based on crystalline phases only. The instrumental peak function was obtained from a measurement of a microcrystalline MgO powder in a 1 mm capillary, i.e., identical to the *in situ* experiment (the LaB_6_ NIST standard in a 1 mm capillary could not be used due to its high absorption at the used energy).

### AIMD Modeling of MgCO_3_ Dissolution

AIMD simulations were performed using a plane-wave basis set, as implemented in VASP.^61,62^ The projector augmented-wave method^63^ was used to describe the core electrons with the Perdew–Burke–Ernzerhof exchange–correlation (XC) functional.^64^ The kinetic energy cutoff for the wave function and charge density was set to 450 eV, and single γ k-point was used. Two MD runs were carried out using the NVT ensemble and a Nose-Hoover thermostat. In the first run, the systems were annealed from 300 to 600 K over 5 ps. In the second run, the resultant final configurations from the first run were annealed at 600 K for another 5 ps. We computed the total energy by taking the average of the final 2 ps of the second run. The solubilities of [Mg^2+^···CO_3_^2–^] ion pairs were investigated in NaNO_3_ and RbNO_3_ molten matrices. The afore-described MD procedure was used to obtain the total energies of the systems with and without the [Mg^2+^···CO_3_^2–^] ion pairs dissolved in NaNO_3_ or RbNO_3_. The Packmol code was used to build these configurations using a unit cell with a size of 10 × 10 × 10 Å.^65^

## Supporting Information Available

Example Rietveld refinements and additional details of the *in situ* XRD setup and the Rietveld refinements. Supplementary results include *in situ* XRD data, (S)TEM and EDX images, TGA results and *ex situ* XRD patterns (PDF)

## References

[ref1] Gabrielli P.; Gazzani M.; Mazzotti M. The Role of Carbon Capture and Utilization, Carbon Capture and Storage, and Biomass to Enable a Net-Zero-CO_2_ Emissions Chemical Industry. Ind. Eng. Chem. Res. 2020, 59 (15), 7033–7045. 10.1021/acs.iecr.9b06579.

[ref2] Paltsev S.; Morris J.; Kheshgi H.; Herzog H. Hard-to-Abate Sectors: The Role of Industrial Carbon Capture and Storage (CCS) in Emission Mitigation. Appl. Energy 2021, 300, 117322 10.1016/j.apenergy.2021.117322.

[ref3] Bui M.; Adjiman C. S.; Bardow A.; Anthony E. J.; Boston A.; Brown S.; Fennell P. S.; Fuss S.; Galindo A.; Hackett L. A.; Hallett J. P.; Herzog H. J.; Jackson G.; Kemper J.; Krevor S.; Maitland G. C.; Matuszewski M.; Metcalfe I. S.; Petit C.; Puxty G.; Reimer J.; Reiner D. M.; Rubin E. S.; Scott S. A.; Shah N.; Smit B.; Trusler J. P. M.; Webley P.; Wilcox J.; Mac Dowell N. Carbon Capture and Storage (CCS): The Way Forward. Energy Environ. Sci. 2018, 11 (5), 1062–1176. 10.1039/C7EE02342A.

[ref4] Choi S.; Drese J. H.; Jones C. W. Adsorbent Materials for Carbon Dioxide Capture from Large Anthropogenic Point Sources. ChemSusChem 2009, 2 (9), 796–854. 10.1002/cssc.200900036. 19731282

[ref5] Rochelle G. T. Amine Scrubbing for CO_2_ Capture. Science 2009, 325 (5948), 1652–1654. 10.1126/science.1176731. 19779188

[ref6] Gao W.; Liang S.; Wang R.; Jiang Q.; Zhang Y.; Zheng Q.; Xie B.; Toe C. Y.; Zhu X.; Wang J.; Huang L.; Gao Y.; Wang Z.; Jo C.; Wang Q.; Wang L.; Liu Y.; Louis B.; Scott J.; Roger A. C.; Amal R.; He H.; Park S. E. Industrial Carbon Dioxide Capture and Utilization: State of the Art and Future Challenges. Chem. Soc. Rev. 2020, 49 (23), 8584–8686. 10.1039/D0CS00025F. 33073812

[ref7] Meng F.; Meng Y.; Ju T.; Han S.; Lin L.; Jiang J. Research Progress of Aqueous Amine Solution for CO_2_ Capture: A Review. Renewable and Sustainable Energy Rev. 2022, 168, 112902 10.1016/j.rser.2022.112902.

[ref8] Buvik V.; Høisæter K. K.; Vevelstad S. J.; Knuutila H. K. A Review of Degradation and Emissions in Post-Combustion CO_2_ Capture Pilot Plants. Int. J. Greenhouse Gas Control 2021, 106, 103246 10.1016/j.ijggc.2020.103246.

[ref9] Dunstan M. T.; Donat F.; Bork A. H.; Grey C. P.; Müller C. R. CO_2_ Capture at Medium to High Temperature Using Solid Oxide-Based Sorbents: Fundamental Aspects, Mechanistic Insights, and Recent Advances. Chem. Rev. 2021, 121 (20), 12681–12745. 10.1021/acs.chemrev.1c00100. 34351127

[ref10] Hu Y.; Guo Y.; Sun J.; Li H.; Liu W. Progress in MgO Sorbents for Cyclic CO_2_ Capture: A Comprehensive Review. J. Mater. Chem. A 2019, 7 (35), 20103–20120. 10.1039/C9TA06930E.

[ref11] Chang R.; Wu X.; Cheung O.; Liu W. Synthetic Solid Oxide Sorbents for CO_2_ Capture: State-of-the Art and Future Perspectives. J. Mater. Chem. A 2022, 10 (4), 1682–1705. 10.1039/D1TA07697C.

[ref12] Kierzkowska A. M.; Pacciani R.; Müller C. R. CaO-Based CO_2_ Sorbents: From Fundamentals to the Development of New, Highly Effective Materials. ChemSusChem 2013, 6 (7), 1130–1148. 10.1002/cssc.201300178. 23821467

[ref13] Martínez A.; Lara Y.; Lisbona P.; Romeo L. M. Energy Penalty Reduction in the Calcium Looping Cycle. Int. J. Greenhouse Gas Control 2012, 7, 74–81. 10.1016/j.ijggc.2011.12.005.

[ref14] Hwang B. W.; Lim J. H.; Chae H. J.; Ryu H. J.; Lee D.; Lee J. B.; Kim H.; Lee S. C.; Kim J. C. CO_2_ Capture and Regeneration Properties of MgO-Based Sorbents Promoted with Alkali Metal Nitrates at High Pressure for the Sorption Enhanced Water Gas Shift Process. Process Saf. Environ. Prot. 2018, 116, 219–227. 10.1016/j.psep.2018.02.008.

[ref15] Choi D. H.; Lee J. B.; Eom T. H.; Baek J. I.; Jegarl S.; Ryu C. K. Study of MgO-Based Dry Regenerable Sorbent for Sorption Enhanced Water Gas Shift Reaction. Renewable Energy 2013, 54, 144–149. 10.1016/j.renene.2012.08.044.

[ref16] Dal Pozzo A.; Armutlulu A.; Rekhtina M.; Abdala P. M.; Müller C. R. CO_2_ Uptake and Cyclic Stability of MgO-Based CO_2_ Sorbents Promoted with Alkali Metal Nitrates and Their Eutectic Mixtures. ACS Appl. Energy Mater. 2019, 2 (2), 1295–1307. 10.1021/acsaem.8b01852.

[ref17] Harada T.; Simeon F.; Hamad E. Z.; Hatton T. A. Alkali Metal Nitrate-Promoted High-Capacity MgO Adsorbents for Regenerable CO_2_ Capture at Moderate Temperatures. Chem. Mater. 2015, 27 (6), 1943–1949. 10.1021/cm503295g.

[ref18] Zhang K.; Li X. S.; Li W.-Z.; Rohatgi A.; Duan Y.; Singh P.; Li L.; King D. L. Phase Transfer-Catalyzed Fast CO_2_ Absorption by MgO-Based Absorbents with High Cycling Capacity. Adv. Mater. Interfaces 2014, 1 (3), 1400030 10.1002/admi.201400030.

[ref19] Gao W.; Xiao J.; Wang Q.; Li S.; Vasiliades M. A.; Huang L.; Gao Y.; Jiang Q.; Niu Y.; Zhang B.; Liu Y.; He H.; Efstathiou A. M. Unravelling the Mechanism of Intermediate-Temperature CO_2_ Interaction with Molten-NaNO_3_ -Salt-Promoted MgO. Adv. Mater. 2022, 34 (4), 2106677 10.1002/adma.202106677. 34729827

[ref20] Landuyt A.; Kumar P. V.; Yuwono J. A.; Bork A. H.; Donat F.; Abdala P. M.; Müller C. R. Uncovering the CO_2_ Capture Mechanism of NaNO_3_-Promoted MgO by ^18^O Isotope Labeling. JACS Au 2022, 2 (12), 2731–2741. 10.1021/jacsau.2c00461. 36590255 PMC9795564

[ref21] Kwak J. S.; Oh K. R.; Kim K. Y.; Lee J. M.; Kwon Y. U. CO_2_ Absorption and Desorption Characteristics of MgO-Based Absorbent Promoted by Triple Eutectic Alkali Carbonate. Phys. Chem. Chem. Phys. 2019, 21 (37), 20805–20813. 10.1039/C9CP03258D. 31515545

[ref22] Kwak J.-S.; Kim K.-Y.; Oh K.-R.; Kwon Y.-U. Performance Enhancement of All-Solid CO_2_ Absorbent Based on Na_2_CO_3_-Promoted MgO by Using ZrO_2_ Dispersant. Int. J. Greenhouse Gas Control 2019, 81, 38–43. 10.1016/j.ijggc.2018.12.010.

[ref23] Lee C. H.; Mun S.; Lee K. B. Characteristics of Na-Mg Double Salt for High-Temperature CO_2_ Sorption. Chem. Eng. J. 2014, 258, 367–373. 10.1016/j.cej.2014.07.082.

[ref24] Gao W.; Zhou T.; Gao Y.; Louis B.; O’Hare D.; Wang Q. Molten Salts-Modified MgO-Based Adsorbents for Intermediate-Temperature CO_2_ Capture: A Review. J. Energy Chem. 2017, 26 (5), 830–838. 10.1016/j.jechem.2017.06.005.

[ref25] Duan Y.; Zhang K.; Li X. S.; King D. L.; Li B.; Zhao L.; Xiao Y. Ab Initio Thermodynamic Study of the CO_2_ Capture Properties of M_2_CO_3_ (M = Na, K)- and CaCO_3_-Promoted MgO Sorbents Towards Forming Double Salts. Aerosol Air Qual. Res. 2014, 14 (2), 470–479. 10.4209/aaqr.2013.05.0178.

[ref26] Kwak J.-S.; Kim K.-Y.; Yoon J. W.; Oh K.-R.; Kwon Y.-U. Interfacial Interactions Govern the Mechanisms of CO_2_ Absorption and Desorption on A_2_CO_3_-Promoted MgO (A = Na, K, Rb, and Cs) Absorbents. J. Phys. Chem. C 2018, 122 (35), 20289–20300. 10.1021/acs.jpcc.8b04895.

[ref27] Zhang K.; Li X. S.; Chen H.; Singh P.; King D. L. Molten Salt Promoting Effect in Double Salt CO_2_ Absorbents. J. Phys. Chem. C 2016, 120 (2), 1089–1096. 10.1021/acs.jpcc.5b10729.

[ref28] Prashar A. K.; Seo H.; Choi W. C.; Kang N. Y.; Park S.; Kim K.; Min D. Y.; Kim H. M.; Park Y. K. Factors Affecting the Rate of CO_2_ Absorption after Partial Desorption in NaNO_3_-Promoted MgO. Energy Fuels 2016, 30 (4), 3298–3305. 10.1021/acs.energyfuels.5b02909.

[ref29] Ding J.; Yu C.; Lu J.; Wei X.; Wang W.; Pan G. Enhanced CO_2_ Adsorption of MgO with Alkali Metal Nitrates and Carbonates. Appl. Energy 2020, 263, 114681 10.1016/j.apenergy.2020.114681.

[ref30] Wang L.; Zhou Z.; Hu Y.; Cheng Z.; Fang X. Nanosheet MgO-Based CO_2_ Sorbent Promoted by Mixed-Alkali-Metal Nitrate and Carbonate: Performance and Mechanism. Ind. Eng. Chem. Res. 2017, 56 (20), 5802–5812. 10.1021/acs.iecr.7b00483.

[ref31] Chen J.; Duan L.; Donat F.; Müller C. R. Assessment of the Effect of Process Conditions and Material Characteristics of Alkali Metal Salt Promoted MgO-Based Sorbents on Their CO_2_ Capture Performance. ACS Sustainable Chem. Eng. 2021, 9 (19), 6659–6672. 10.1021/acssuschemeng.1c00262.

[ref32] Cui H.; Zhang Q.; Hu Y.; Peng C.; Fang X.; Cheng Z.; Galvita V. V.; Zhou Z. Ultrafast and Stable CO_2_ Capture Using Alkali Metal Salt-Promoted MgO-CaCO_3_ Sorbents. ACS Appl. Mater. Interfaces 2018, 10 (24), 20611–20620. 10.1021/acsami.8b05829. 29855184

[ref33] Jin S.; Ho K.; Vu A. T.; Lee C. H. Salt-Composition-Controlled Precipitation of Triple-Salt-Promoted MgO with Enhanced CO_2_ Sorption Rate and Working Capacity. Energy Fuels 2017, 31 (9), 9725–9735. 10.1021/acs.energyfuels.7b01428.

[ref34] Cherginets V. L.; Deineka T. G.; Demirskaya O. V.; Rebrova T. P. Potentiometric Investigation of Oxide Solubilities in Molten KCl–NaCl Eutectic.: The Effect of Surface Area of Solid Particles on the Solubilities. J. Electroanal. Chem. 2002, 531 (2), 171–178. 10.1016/S0022-0728(02)01061-6.

[ref35] FTsalt - FACT Salt Phase Diagrams (377)

[ref36] Vu A.-T.; Ho K.; Jin S.; Lee C.-H. Double Sodium Salt-Promoted Mesoporous MgO Sorbent with High CO_2_ Sorption Capacity at Intermediate Temperatures under Dry and Wet Conditions. Chem. Eng. J. 2016, 291, 161–173. 10.1016/j.cej.2016.01.080.

[ref37] Rekhtina M.; Krödel M.; Wu Y.-H.; Kierzkowska A.; Donat F.; Abdala P. M.; Müller C. R. Deciphering the Structural Dynamics in Molten Salt–Promoted MgO-Based CO_2_ Sorbents and Their Role in the CO_2_ Uptake. Sci. Adv. 2023, 9 (26), eadg5690 10.1126/sciadv.adg5690. 37379379 PMC10306292

[ref38] Li J.; Deepak F. L. *In Situ* Kinetic Observations on Crystal Nucleation and Growth. Chem. Rev. 2022, 122 (23), 16911–16982. 10.1021/acs.chemrev.1c01067. 36347015

[ref39] Ward M. D. Bulk Crystals to Surfaces: Combining X-Ray Diffraction and Atomic Force Microscopy to Probe the Structure and Formation of Crystal Interfaces. Chem. Rev. 2001, 101 (6), 1697–1726. 10.1021/cr000020j. 11709996

[ref40] Hooks D. E.; Fritz T.; Ward M. D. Epitaxy and Molecular Organization on Solid Substrates. Adv. Mater. 2001, 13 (4), 227–241. 10.1002/1521-4095(200102)13:4<227::AID-ADMA227>3.0.CO;2-P.

[ref41] Jo S.-I.; An Y.-I.; Kim K.-Y.; Choi S.-Y.; Kwak J.-S.; Oh K.-R.; Kwon Y.-U. Mechanisms of Absorption and Desorption of CO_2_ by Molten NaNO_3_ -Promoted MgO. Phys. Chem. Chem. Phys. 2017, 19 (8), 6224–6232. 10.1039/C6CP07787K. 28195289

[ref42] Papalas T.; Polychronidis I.; Antzaras A. N.; Lemonidou A. A. Enhancing the Intermediate-Temperature CO_2_ Capture Efficiency of Mineral MgO via Molten Alkali Nitrates and CaCO_3_: Characterization and Sorption Mechanism. J. CO2 Util. 2021, 50, 101605 10.1016/j.jcou.2021.101605.

[ref43] Biasin A.; Segre C. U.; Strumendo M. CaCO_3_ Crystallite Evolution during CaO Carbonation: Critical Crystallite Size and Rate Constant Measurement by In-Situ Synchrotron Radiation X-Ray Powder Diffraction. Cryst. Growth Des. 2015, 15 (11), 5188–5201. 10.1021/acs.cgd.5b00563.

[ref44] Biasin A.; Segre C. U.; Salviulo G.; Zorzi F.; Strumendo M. Investigation of CaO–CO_2_ Reaction Kinetics by in-Situ XRD Using Synchrotron Radiation.. Chem. Eng. Sci. 2015, 127, 13–24. 10.1016/j.ces.2014.12.058.

[ref45] Bauer S.; Rodrigues A.; Baumbach T. Real Time in Situ X-Ray Diffraction Study of the Crystalline Structure Modification of Ba_0.5_Sr_0.5_TiO_3_ during the Post-Annealing. Sci. Rep. 2018, 8 (1), 11969 10.1038/s41598-018-30392-y. 30097626 PMC6086881

[ref46] Langford J. I.; Louër D.; Scardi P. Effect of a Crystallite Size Distribution on X-Ray Diffraction Line Profiles and Whole-Powder-Pattern Fitting. J. Appl. Crystallogr. 2000, 33 (3), 964–974. 10.1107/S002188980000460X.

[ref47] Gao W.; Vasiliades M. A.; Damaskinos C. M.; Zhao M.; Fan W.; Wang Q.; Reina T. R.; Efstathiou A. M. Molten Salt-Promoted MgO Adsorbents for CO_2_ Capture: Transient Kinetic Studies. Environ. Sci. Technol. 2021, 55 (8), 4513–4521. 10.1021/acs.est.0c08731. 33749277

[ref48] Khawam A.; Flanagan D. R. Solid-State Kinetic Models: Basics and Mathematical Fundamentals. J. Phys. Chem. B 2006, 110 (35), 17315–17328. 10.1021/jp062746a. 16942065

[ref49] German R. M.; Suri P.; Park S. J. Review: Liquid Phase Sintering. J. Mater. Sci. 2009, 44 (1), 1–39. 10.1007/s10853-008-3008-0.

[ref50] Temple R. B.; Lockyer C. J. Solubility Data for Na_2_CO_3_ and K_2_CO_3_ Dissolved in Molten NaNO_3_/KNO_3_ Eutectic. Aust. J. Chem. 1979, 32 (8), 1849–1850. 10.1071/CH9791849.

[ref51] Paun C.; Safonova O. V.; Szlachetko J.; Abdala P. M.; Nachtegaal M.; Sa J.; Kleymenov E.; Cervellino A.; Krumeich F.; van Bokhoven J. A. Polyhedral CeO_2_ Nanoparticles: Size-Dependent Geometrical and Electronic Structure. J. Phys. Chem. C 2012, 116 (13), 7312–7317. 10.1021/jp300342b.

[ref52] Cimino A.; Porta P.; Valigi M. Dependence of the Lattice Parameter of Magnesium Oxide on Crystallite Size. J. Am. Ceram. Soc. 1966, 49 (3), 152–156. 10.1111/j.1151-2916.1966.tb15394.x.

[ref53] Tóth G. I.; Tegze G.; Pusztai T.; Gránásy L. Heterogeneous Crystal Nucleation: The Effect of Lattice Mismatch. Phys. Rev. Lett. 2012, 108 (2), 025502 10.1103/PhysRevLett.108.025502. 22324697

[ref54] Li L.; Fijneman A. J.; Kaandorp J. A.; Aizenberg J.; Noorduin W. L. Directed Nucleation and Growth by Balancing Local Supersaturation and Substrate/Nucleus Lattice Mismatch. Proc. Natl. Acad. Sci. U.S.A 2018, 115 (14), 3575–3580. 10.1073/pnas.1712911115. 29555753 PMC5889625

[ref55] Karoui N. K.; Hellali D.; Saidi A.; Zamali H. The Phase Diagram of the Isobaric Binary System (NaNO_3_ + RbNO_3_). J. Therm. Anal. Calorim. 2016, 124 (3), 1145–1151. 10.1007/s10973-015-5226-4.

[ref56] Wang T.; Xu A.; Cölfen H. Formation of Self-Organized Dynamic Structure Patterns of Barium Carbonate Crystals in Polymer-Controlled Crystallization. Angew. Chem., Int. Ed. 2006, 45 (27), 4451–4455. 10.1002/anie.200601038. 16789037

[ref57] De Yoreo J. J.; Vekilov P. G. Principles of Crystal Nucleation and Growth. Rev. Mineral. Geochem. 2003, 54 (1), 57–93. 10.2113/0540057.

[ref58] Cui H.; Cheng Z.; Zhou Z. Unravelling the Role of Alkaline Earth Metal Carbonates in Intermediate Temperature CO_2_ Capture Using Alkali Metal Salt-Promoted MgO-Based Sorbents. J. Mater. Chem. A 2020, 8 (35), 18280–18291. 10.1039/D0TA06170K.

[ref59] Dyadkin V.; Pattison P.; Dmitriev V.; Chernyshov D. A New Multipurpose Diffractometer PILATUS@SNBL. J. Synchrotron Radiat. 2016, 23 (3), 825–829. 10.1107/S1600577516002411. 27140164

[ref60] Coelho A. A. *TOPAS* and *TOPAS-Academic* : An Optimization Program Integrating Computer Algebra and Crystallographic Objects Written in C++. J. Appl. Crystallogr. 2018, 51 (1), 210–218. 10.1107/S1600576718000183.

[ref61] Kresse G.; Furthmüller J. Efficient Iterative Schemes for Ab Initio Total-Energy Calculations Using a Plane-Wave Basis Set. Phys. Rev. B 1996, 54 (16), 11169–11186. 10.1103/PhysRevB.54.11169. 9984901

[ref62] Kresse G.; Furthmüller J. Efficiency of Ab-Initio Total Energy Calculations for Metals and Semiconductors Using a Plane-Wave Basis Set. Comput. Mater. Sci. 1996, 6 (1), 15–50. 10.1016/0927-0256(96)00008-0.

[ref63] Kresse G.; Joubert D. From Ultrasoft Pseudopotentials to the Projector Augmented-Wave Method. Phys. Rev. B 1999, 59 (3), 1758–1775. 10.1103/PhysRevB.59.1758.

[ref64] Perdew J. P.; Burke K.; Ernzerhof M. Generalized Gradient Approximation Made Simple. Phys. Rev. Lett. 1996, 77 (18), 3865–3868. 10.1103/PhysRevLett.77.3865. 10062328

[ref65] Martínez L.; Andrade R.; Birgin E. G.; Martínez J. M. PACKMOL: A Package for Building Initial Configurations for Molecular Dynamics Simulations. J. Comput. Chem. 2009, 30 (13), 2157–2164. 10.1002/jcc.21224. 19229944

