# Supplementary material for: Role of Na2CO3 as Nucleation Seeds to Accelerate the CO2 Uptake Kinetics of MgO-Based Sorbents
Source: JACS Au. 2024 Nov 16;4(12):4809–20. doi: 10.1021/jacsau.4c00782 (PMC11672151; doi:10.1021/jacsau.4c00782)
Supplement: Supplementary file 1 — au4c00782_si_001.pdf [file au4c00782_si_001.pdf]

Supporting Information for

The Role of Na_2CO_3 as Nucleation Seeds to Accelerate the CO_2 Uptake Kinetics of MgO-Based Sorbents

Annelies Landuyt[a], Ilia Kochetygov[b], Charles J. McMonagle[c], Priyank V. Kumar[d], Jodie A. Yuwono[e], Wendy L. Queen[f], Paula M. Abdala[a*] and Christoph R. Müller[a]*

[a] Laboratory of Energy Science and Engineering, Department of Mechanical and Process Engineering, Eidgenössische Technische Hochschule (ETH) Zürich, 8092 Zürich, Switzerland

[b] Paul Scherrer Institut, PSI Center for Energy and Environmental Sciences, Villigen PSI CH-5232, Switzerland

[c] Swiss-Norwegian Beamlines (SNBL), European Synchrotron Radiation Facility (ESRF), Grenoble 38000, France

[d] School of Chemical Engineering, The University of New South Wales (UNSW Sydney), 2052 Sydney New South Wales, Australia

[e] School of Chemical Engineering, The University of Adelaide, Adelaide, SA 5005, Australia

[f] Institute of Chemical Sciences and Engineering (ISIC), École Polytechnique Fédérale de Lausanne (EPFL), 1051 Sion, Switzerland

* Corresponding authors:

Dr. Paula M. Abdala, e-mail: abdalap@ethz.ch

Prof. Dr. Christoph R. Müller, email: muelchri@ethz.ch

Table of Contents

Characterization of the as-synthesized materials... 3

Optimization of the amount of Na_2CO_3 in MgO-(Na_2CO_3/$NaNO_3$).. 4

Selected XRD patterns of MgO-$NaNO_3$ and MgO-(Na_2CO_3/$NaNO_3$)... 5

Examples of Rietveld refinements.. 6

Theoretical volume expansions upon converting MgO into $MgCO_3$ for two possible mechanisms....... 8

CO_2 uptake curves obtained in a TGA for MgO-$NaNO_3$ and MgO-(Na_2CO_3/$NaNO_3$)........................... 9

The Na_2CO_3 phase evolution in MgO-(Na_2CO_3/$NaNO_3$).. 11

Illustration of sintering during $MgCO_3$ formation... 12

TEM-EDX analysis 13

Cyclic CO_2 uptake for MgO-(Na_2CO_3/$NaNO_3$) and MgO-$NaNO_3$ recorded in a TGA 15

The $MgCO_3$ lattice parameters as a function of MgO conversion.. 16

The lattice parameters of $MgCO_3$ as a function of the average $MgCO_3$ crystallite size........................ 17

Evolution of the lattice parameter of MgO during carbonation 18

$RbNO_3$ promotion: CO_2 uptake curves obtained in a TGA 19

Selected XRD patterns collected during the carbonation of (Na_2CO_3/$RbNO_3$)-promoted MgO 20

Selected Rietveld refinements for (Na_2CO_3/$RbNO_3$)-promoted MgO 21

In situ XRD data during the carbonation of MgO-Na_2CO_3.. 22

Calculation of the dissolution energy of $MgCO_3$ in $RbNO_3$... 23

The *in situ* XRD setup 24

Characterization of the as-synthesized materials

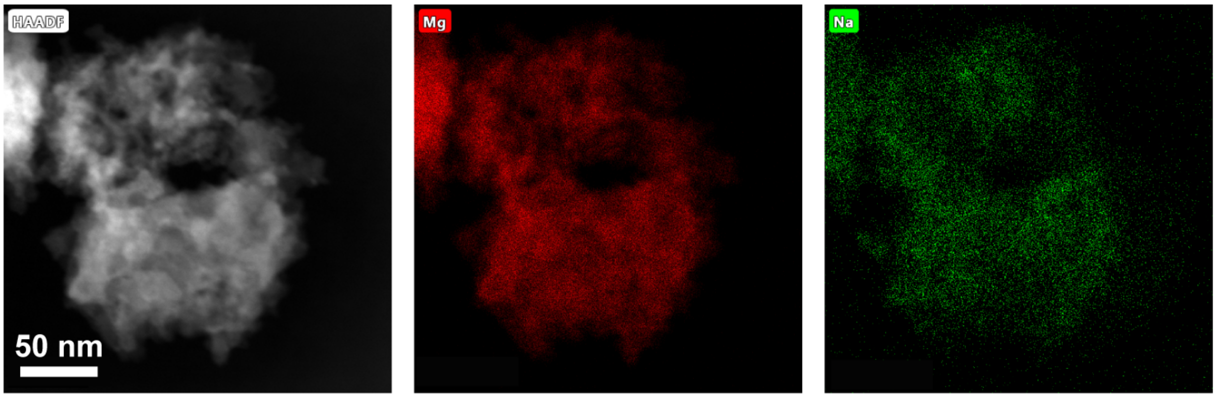

Figure S1. STEM-EDX maps of ball-milled MgO-Na_2CO_3 (a) high-angle annular dark field-scanning transmission electron microscopy (HAADF-STEM) image, (b) Mg signal and (c) Na signal.

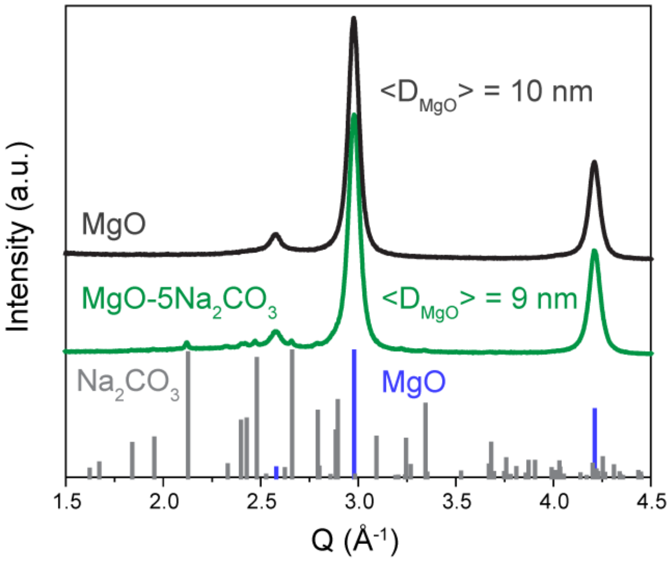

Figure S2. Laboratory-based (Cu Kα-radiation) XRD patterns of as-synthesized MgO and MgO-Na_2CO_3 overlaid with calculated intensity references for MgO and Na_2CO_3.

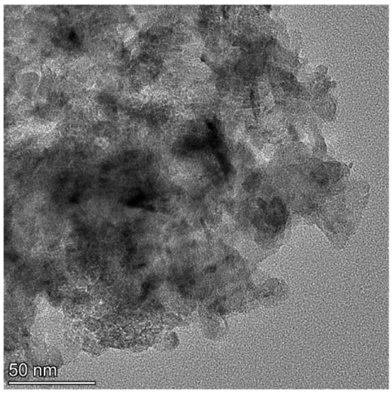

Figure S3. TEM image of ball milled MgO.

Optimization of the amount of Na_2CO_3 in MgO-(Na_2CO_3/$NaNO_3$)

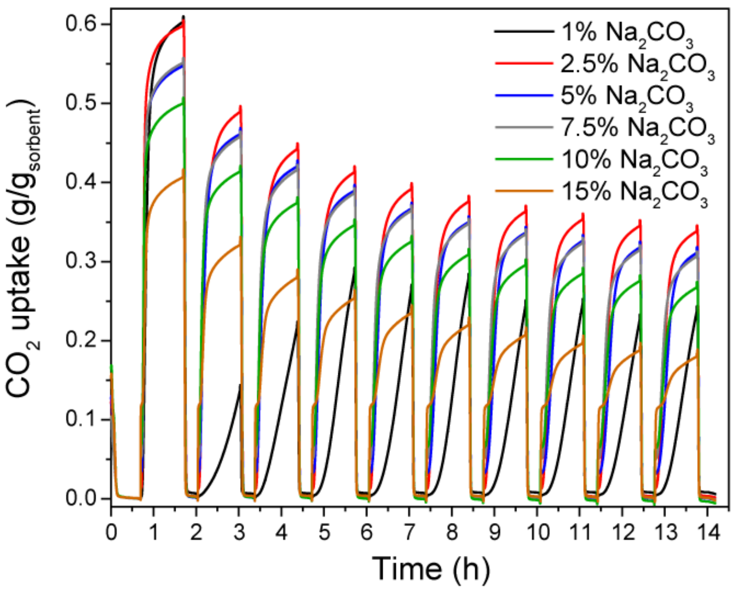

Figure S4. Cyclic CO_2 uptake observed in a TGA for MgO promoted with different mol% Na_2CO_3 and 10 mol% $NaNO_3$ over 10 cycles of CO_2 uptake (1 h at 315 °C in CO_2) and regeneration (15 min at 450 °C in N_2).

Selected XRD patterns of MgO-NaNO$_3$ and MgO-(Na$_2$CO$_3$/NaNO$_3$)

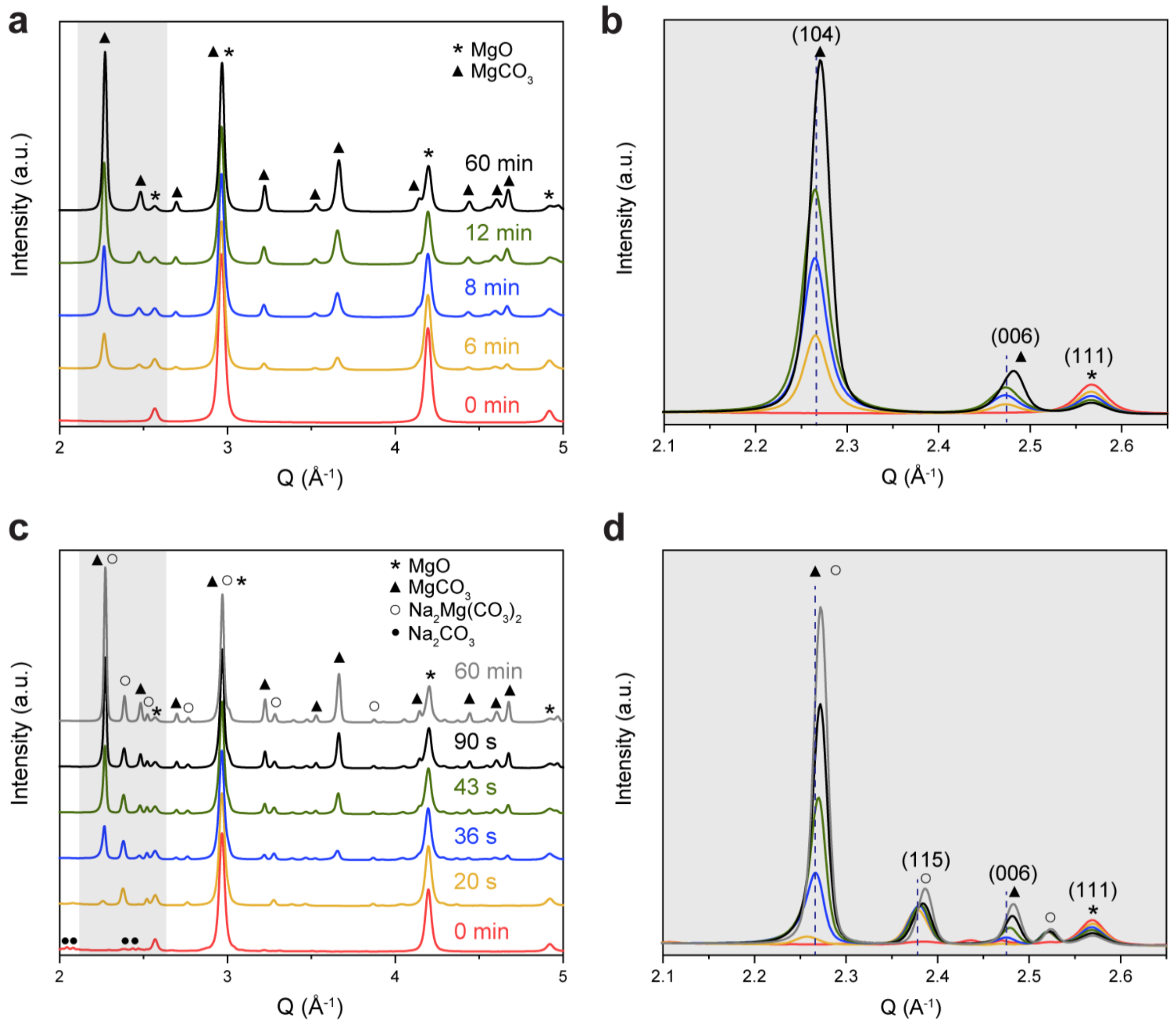

Figure S5. Stacked XRD patterns at various points in time during the carbonation reaction of (a, b) MgO-NaNO$_3$ and (c, d) MgO-(Na$_2$CO$_3$/NaNO$_3$).

Examples of Rietveld refinements

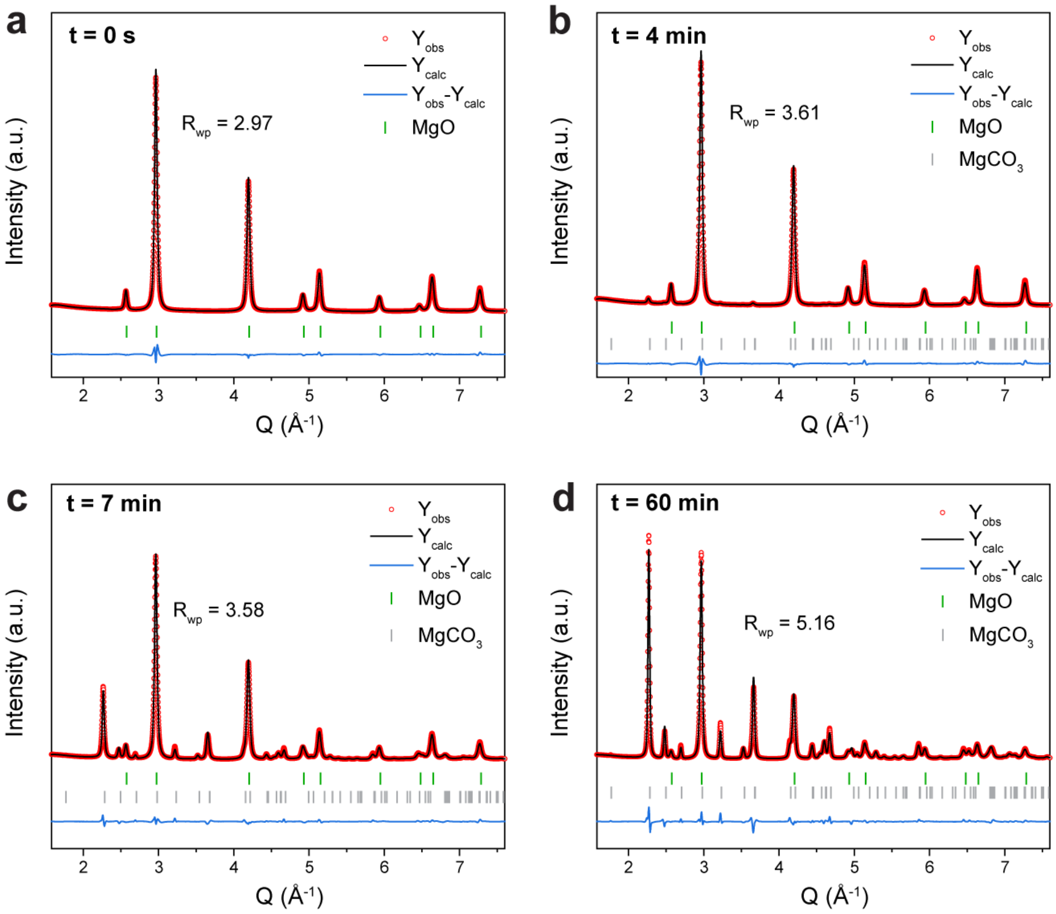

Figure S6. Example Rietveld refinements on patterns obtained at different points in time during the carbonation reaction of MgO-$NaNO_3$: (a) t = 0, (b) t = 4 min, (c) t = 7 min and (d) t = 60 min. (λ = 0.69668 Å)

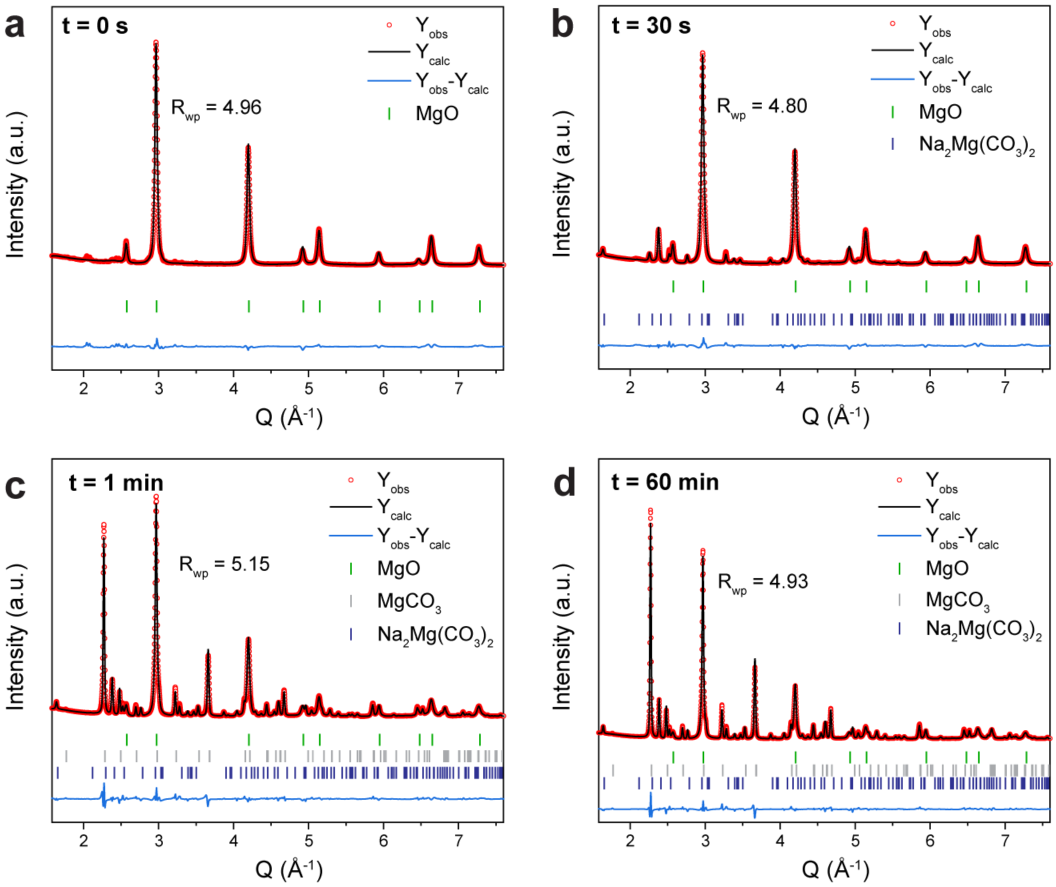

Figure S7. Example Rietveld refinements of MgO-(Na_2CO_3/$NaNO_3$) at different points in time during the carbonation reaction: (a) t = 0, (b) t = 30 s, (c) t= 1 min and (d) t = 60 min. (λ = 0.69668 Å)

Theoretical volume expansions upon converting MgO into $MgCO_3$ for two possible mechanisms

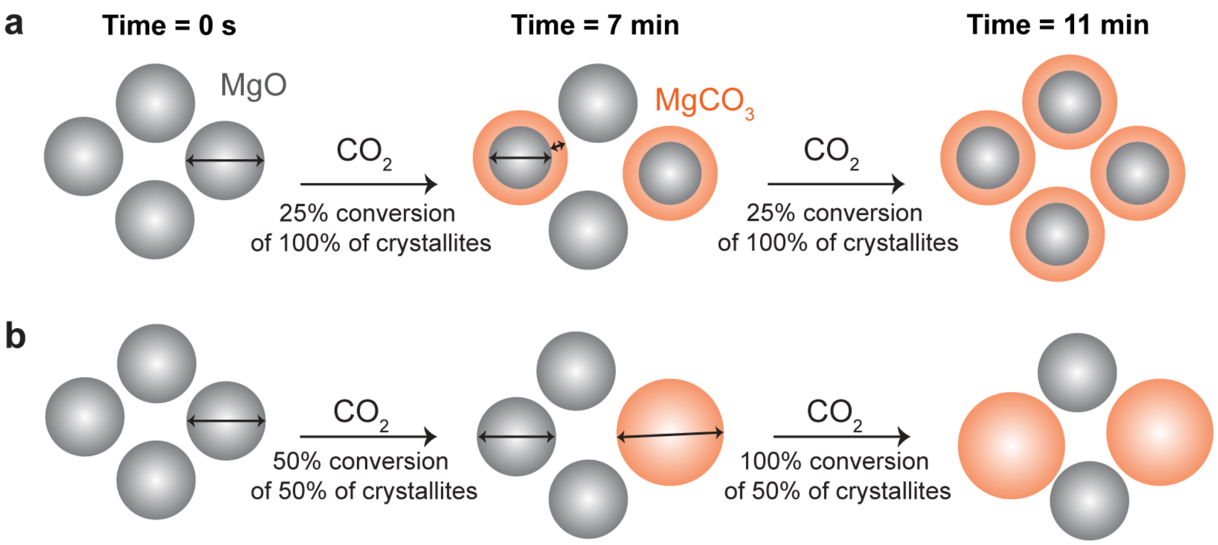

Figure S8: Illustration of two possible conversion mechanisms of MgO crystallites into $MgCO_3$ at 50% conversion. (a) Mechanism 1: a $MgCO_3$ product layer forms on all MgO particles, leading to a reduction in the MgO crystallite size. (b) Mechanism 2: half of the MgO crystallites fully convert into $MgCO_3$, while the other half remains unchanged (e.g. due to an inaccessibility to CO_2). Here, the MgO crystallite size remains constant during the reaction.

The evolution of the crystallite sizes of MgO and $MgCO_3$ during the carbonation reaction provides insight into the growth mechanism of $MgCO_3$. For MgO-$NaNO_3$, The $MgCO_3$ average crystallite size increases to 23 nm during the early growth phase (Stage II.1), however, in the subsequent growth phase (Stage II.2), the $MgCO_3$ average crystallite size remains constant. Indeed, throughout the entire carbonation reaction, the MgO average crystallite size remains unchanged at 17 nm, despite significant changes in the amount of MgO, viz. > 50% MgO conversion. Figure S8 gives an idea about the expected sizes of MgO and $MgCO_3$ for two conceivable reaction mechanisms. The first mechanism assumes that $MgCO_3$ grows as a layer on top of MgO, similar to the proposed growth mechanism of $CaCO_3$ onto CaO.[1] The second mechanism assumes that some MgO crystallites are fully converted into $MgCO_3$ while others remain unreacted. This could be e.g. due to an uneven distribution of the promoter $NaNO_3$ and the MgO crystallites because of agglomeration (several crystallites composing a larger particle as illustrated in the main manuscript Figure 1d).

Assuming spherical crystallites of MgO with a size of 17 nm and considering the molar expansion factor of 252% when converting MgO (molar volume of 11.3 cm^3 mol^{-1}) into $MgCO_3$ (28.5 cm^3 mol^{-1})), the crystallite sizes for MgO (D_{MgO}) and $MgCO_3$ (D_{MgCO3}) in the carbonated material (at 50% MgO conversion) for the two mechanisms in Figure S8 can be estimated as:

- Mechanism 1: D_{MgO} = 13.5 nm and D_{MgCO3} = 7 nm
- Mechanism 2: D_{MgO} = 17 nm and D_{MgCO3} = 23 nm

Scenario 2 aligns with the observed results for MgO-$NaNO_3$. The molar volumes are calculated based on the crystal structures of MgO (ICSD-101007) and $MgCO_3$ (ICSD-40119) from the ICSD database.

CO_2 uptake curves obtained in a TGA for MgO-$NaNO_3$ and MgO-(Na_2CO_3/$NaNO_3$)

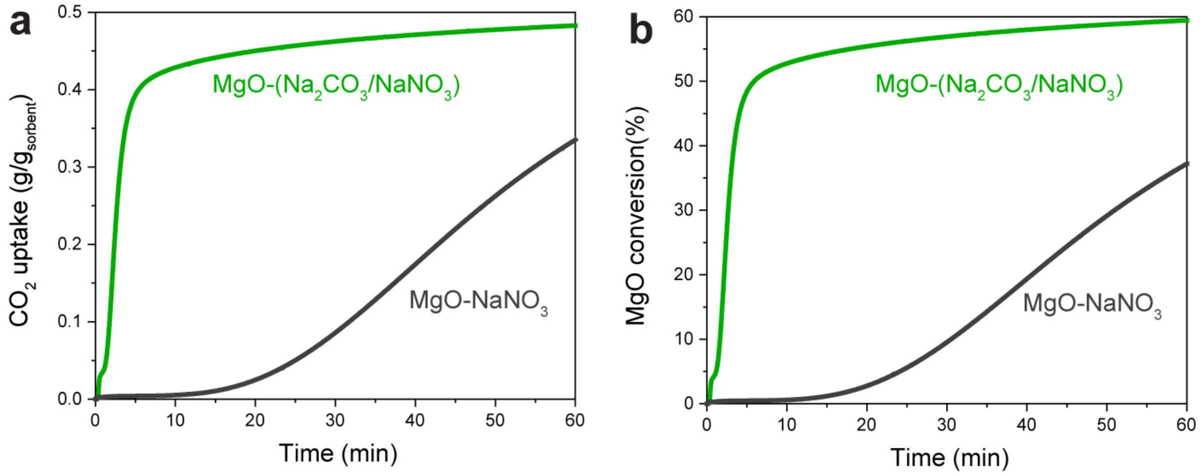

Figure S9. (a) CO_2 uptake and (b) MgO conversion as obtained by TGA for MgO-(Na_2CO_3/$NaNO_3$) (green) and MgO-$NaNO_3$ (black). The CO_2 uptake after 1 h of carbonation for MgO-$NaNO_3$ and MgO-(Na_2CO_3/$NaNO_3$) is 0.33 g $g_{sorbent}^{-1}$ and 0.48 g $g_{sorbent}^{-1}$, respectively. Compared to the CO_2 uptake determined based on the *in situ* XRD measurements (after 1 h of carbonation), i.e. 0.52 g $g_{sorbent}^{-1}$ and 0.45 g $g_{sorbent}^{-1}$ for MgO-$NaNO_3$ and MgO-(Na_2CO_3/$NaNO_3$), respectively, the results are similar. However, the CO_2 uptake of MgO-$NaNO_3$ is significantly higher in the *in situ* XRD experiment, most likely due to the specifics of the setup used. In the *in situ* XRD experiment, the CO_2 flow passes through the sample, whereas in the TGA, CO_2 flows over the sample, resulting in a lower mass transfer to the sample in the TGA.[2] Furthermore, there is a higher space velocity in the *in situ* XRD experiment as compared to the TGA experiments (6.75 mL min^{-1} mg^{-1} in *in situ* XRD and 5.33 mL min^{-1} mg^{-1} in the TGA).

The MgO conversion as a function of the carbonation time t (denoted as C (t)) is calculated based on the weight change observed in the TGA as follows:

$$C\,(t) = \frac{n_{MgO}(t) - n_{MgO}\,(t = 0)}{n_{MgO}\,(t = 0)} \cdot 100\% = \frac{n_{CO2,captured}(t)}{n_{MgO}\,(t = 0)} \qquad (S1)$$

Where $n_{MgO}(t)$ is the number of moles of MgO at a given time t and $n_{CO2,captured}(t)$ is the number of moles of CO_2 captured at a given time t. $n_{CO2,captured}$ is equivalent to the number of moles of MgO that are converted into $MgCO_3$ or $Na_2Mg(CO_3)_2$:

$$MgO + CO_2 \rightleftharpoons MgCO_3 \qquad (S2)$$

$$Na_2CO_3 + MgO + CO_2 \rightleftharpoons Na_2Mg(CO_3)_2 \qquad (S3)$$

Equation S1 can also be expressed as:

$$C\,(t) = \frac{\dfrac{m_{sorbent}(t) - m_{sorbent}(t = 0)}{MW_{CO2}}}{\omega_{MgO} \cdot \dfrac{m_{sorbent}(t = 0)}{MW_{MgO}}} \cdot 100\% \qquad (S4)$$

where:

- $m_{sorbent}(t)$ is the weight of the sorbent at a given time t

- MW is the molecular weight
- ω_{MgO}, is the mass fraction of MgO in the sorbent at time $t = 0$, which can be expressed as :

$$\omega_{MgO} = \frac{mass\ of\ MgO}{total\ mass\ of\ sorbent} = \frac{n_{MgO} \cdot MW_{MgO}}{n_{MgO} \cdot MW_{MgO} + n_{p1} \cdot MW_{p1} + n_{p2} \cdot MW_{p2}} \qquad (S5)$$

where n_{p1} and n_{p2} are, respectively, the molar fractions of promoter 1 and promoter 2 with respect to MgO, and n_{MgO} is 1. For example, for MgO (MW = 40.3 g mol^{-1}) promoted with 10 mol% $NaNO_3$ (MW = 84.99 g mol^{-1}) and 5 mol% Na_2CO_3 (MW = 105.99 g mol^{-1}), we obtain:

$$\omega_{MgO} = \frac{40.3}{40.3 + 0.1 \cdot 84.99 + 0.05 \cdot 105.99} = 0.74$$

And the CO_2 uptake as a function of the carbonation time t is determined using the following formula:

$$CO_2\ uptake\ (t) = \frac{m_{sorbent}(t) - m_{sorbent}(t = 0)}{m_{sorbent}(t = 0)} \qquad (S6)$$

The Na_2CO_3 phase evolution in MgO-(Na_2CO_3/$NaNO_3$)

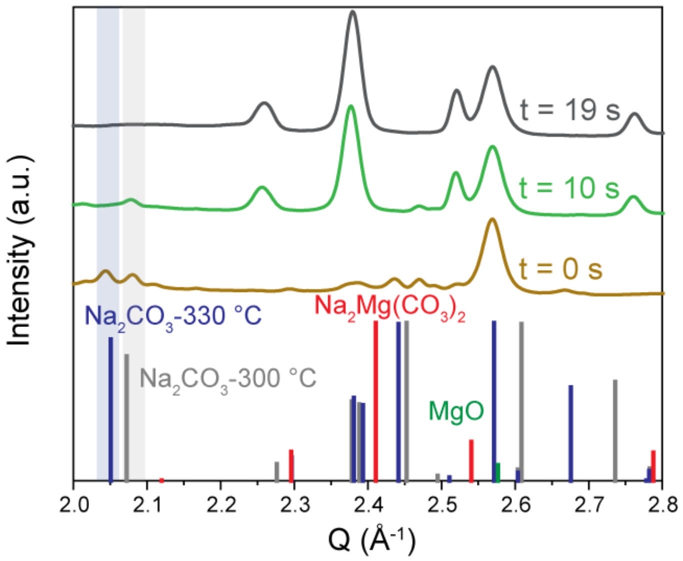

Figure S10. Stacked XRD patterns obtained during the carbonation reaction of MgO-(Na_2CO_3/$NaNO_3$) at time = 0 s, 10 s and 19 s, overlaid with reference patterns for γ-Na_2CO_3 (both at 330 °C (ICSD-80985) and at 300 °C (ICSD-149659)), MgO (ICSD-101007) and $Na_2Mg(CO_3)_2$. The blue shading highlights a representative peak of γ-Na_2CO_3 (ICSD-80985), while the grey shading highlights a representative peak of γ-Na_2CO_3 (ICSD-149659). It can be seen that most of Na_2CO_3 is converted into $Na_2Mg(CO_3)_2$ after 10 seconds of carbonation and no crystalline Na_2CO_3 can be detected after 19 seconds of carbonation, which corresponds to the end of stage I, as indicated in Figure 1f of the main manuscript.

Illustration of sintering during $MgCO_3$ formation

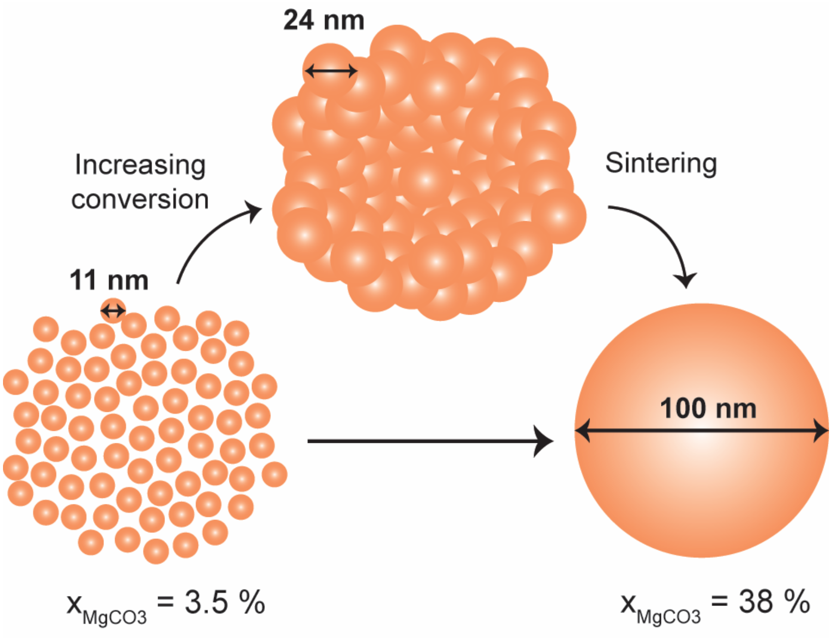

Figure S11. Illustration of the increase in the average $MgCO_3$ crystallite size during the carbonation of MgO-(Na_2CO_3/$NaNO_3$), showing that the increase in crystallite size results from both an increase in the amount of $MgCO_3$ formed and the sintering of $MgCO_3$.

For MgO-(Na_2CO_3/$NaNO_3$), the increase in the average $MgCO_3$ crystallite size during the carbonation cannot be explained solely by the increase in the amount of $MgCO_3$ formed, as illustrated in Figure S11. At the start of the rapid $MgCO_3$ growth stage (Stage II.2), the molar fraction of $MgCO_3$ is 3.5% and the average $MgCO_3$ crystallite size is 11 nm. By the end of stage II.2, the molar fraction of $MgCO_3$ is 38%, and the average $MgCO_3$ crystallite size is 100 nm. However, the tenfold increase in the amount of $MgCO_3$ (from 3% to 38%) would result in an increase in the average diameter of the $MgCO_3$ crystallites by a factor of ~ 2.15 ($\sqrt[3]{10}$). Therefore, the observed increase in the average $MgCO_3$ crystallite size is due to both an increased amount of $MgCO_3$ formed and $MgCO_3$ sintering.

TEM-EDX analysis

Methanol washing step to remove $NaNO_3$ prior to TEM-EDX analysis

The carbonated Na_2CO_3-$NaNO_3$-promoted MgO contains Na in the form of $Na_2Mg(CO_3)_2$ and $NaNO_3$. This complicates the interpretation of the EDX data. More specifically, we are interested in how $Na_2Mg(CO_3)_2$ is distributed in the material and how it compares to the distribution of Na_2CO_3 in the as-synthesized material. Therefore, we have performed a washing step with methanol to selectively remove $NaNO_3$. XRD patterns of the sample before and after the washing show that $NaNO_3$ is completely removed, while the other phases (MgO, $MgCO_3$ and $Na_2Mg(CO_3)_2$) remain intact, see Figure S12.

Washing procedure:

- 10 mg sample was dispersed in 2 mL methanol at room temperature, followed by centrifugation at 6000 rpm for 2 min to precipitate the sample. The supernatant containing the dissolved $NaNO_3$ was discarded. This washing step was repeated twice to ensure that $NaNO_3$ was completely removed. Finally, the material was dried on a hotplate at 80 °C.

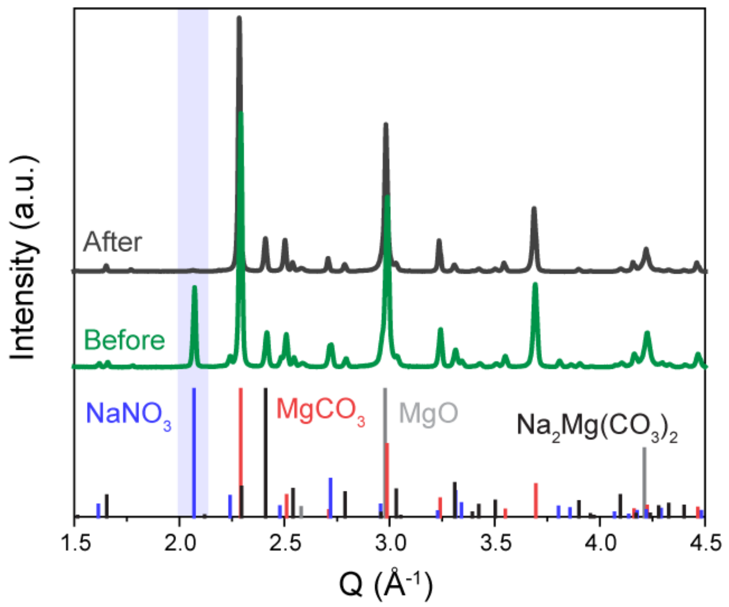

Figure S12. Laboratory-based (Cu Kα-radiation) XRD patterns of (Na_2CO_3/$NaNO_3$)-MgO after 1 h of carbonation at 315 °C in CO_2 (in a TGA) prior to and after washing with methanol, in overlay with the reference patterns for $NaNO_3$, $MgCO_3$, MgO and $Na_2Mg(CO_3)_2$. The blue shading highlights the position of the main peak of $NaNO_3$, clearly indicating that $NaNO_3$ is removed during the washing step. The washing step did not affect the other phases.

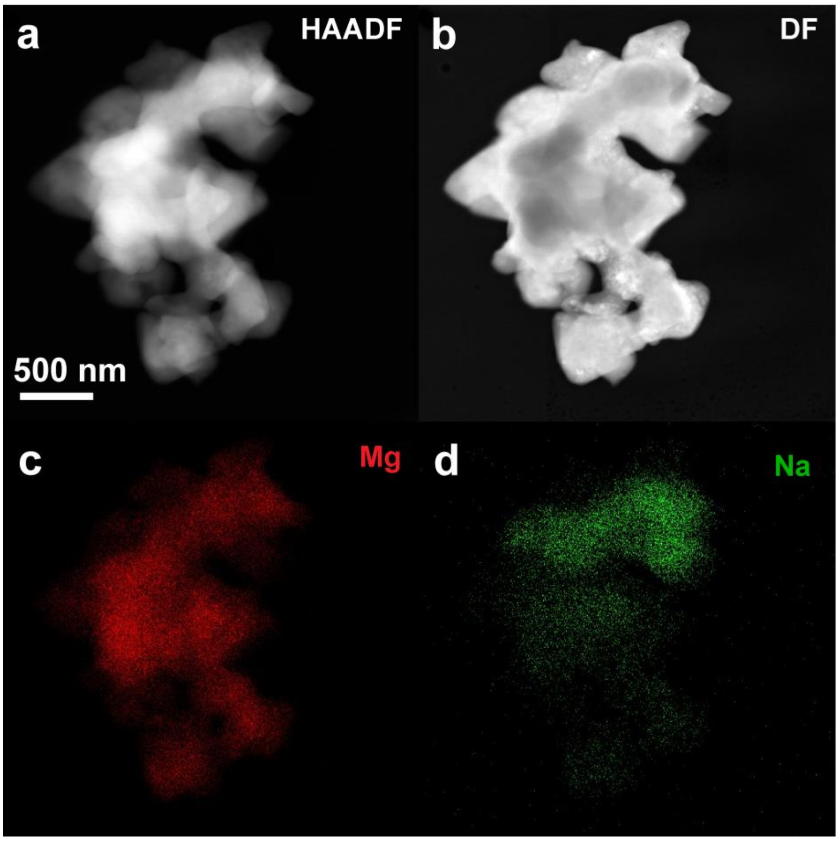

Figure S13. STEM-EDX analysis of MgO-(Na_2CO_3/$NaNO_3$) after 1 h of carbonation at 315 °C in CO_2 in a TGA and after the removal of $NaNO_3$ by washing with methanol (see Figure S12 for more details). (a) High-angle annular dark-field (HAADF) image, (b) dark-field (DF) image and corresponding EDX maps of (c) Mg and (d) Na. The HAADF image reveals the presence of large particles, consistent with *in situ* XRD data (indicating sintering), presented in Figure 1g of the main manuscript. The DF image indicates that, in addition to the large $MgCO_3$ particles seen in the HAADF image, smaller $MgCO_3$ particles are also present. The Na and Mg EDX maps show that Na (a Na containing phase) is partially segregated.

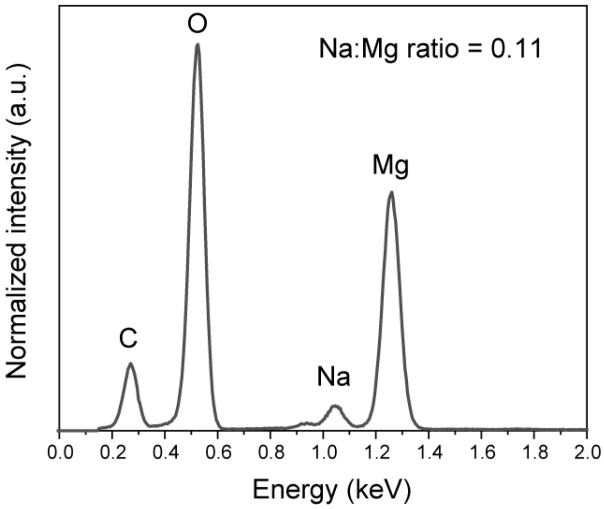

Figure S14. EDX spectrum corresponding to the area shown in Figure S13. The Na:Mg ratio is 0.11, which is very close to the Na:Mg ratio of 0.09 in the as-prepared sorbent as determined by ICP.

Cyclic CO_2 uptake for MgO-(Na_2CO_3/$NaNO_3$) and MgO-$NaNO_3$ recorded in a TGA

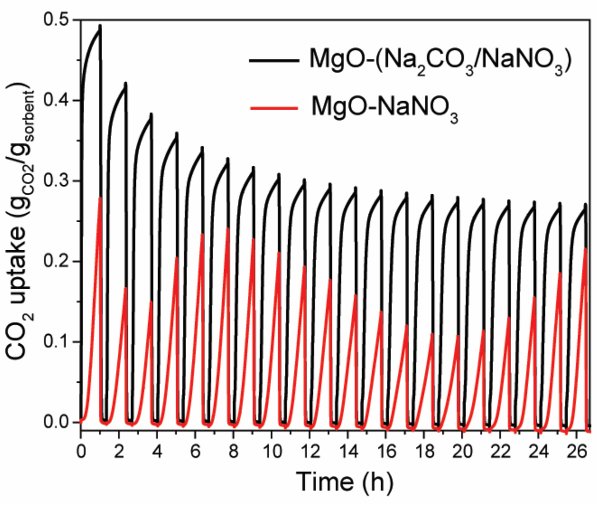

Figure S15. Cyclic CO_2 uptake data obtained by TGA for MgO-(Na_2CO_3/$NaNO_3$) (black) and MgO-$NaNO_3$ (red) during 20 cycles of carbonation and regeneration. The carbonation is performed in CO_2 for 1h at 315 °C and the regeneration in N_2 at 450 °C for 15 min.

The $MgCO_3$ lattice parameters as a function of MgO conversion

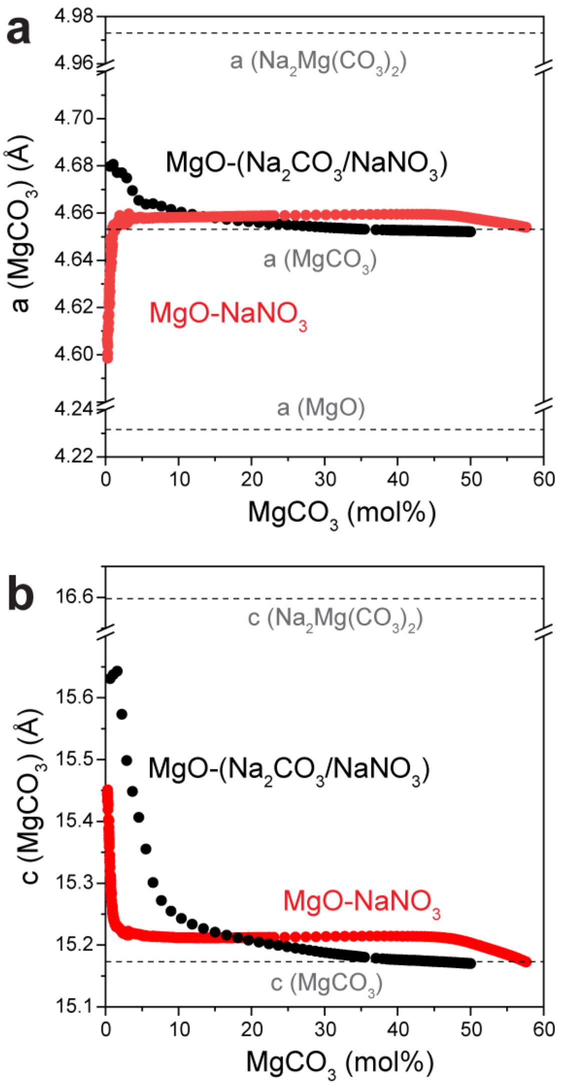

Figure S16. Evolution of (a) the a-parameter and (b) the c-parameter of $MgCO_3$ during the carbonation of MgO-$NaNO_3$ (red dots) and MgO-(Na_2CO_3/$NaNO_3$) (black dots) as a function of $MgCO_3$ formation. The respective equilibrium values (values obtained after 60 min of carbonation) of the lattice parameters for MgO, $MgCO_3$ and $Na_2Mg(CO_3)_2$ are included in the plot (grey dashed lines).

The lattice parameters of $MgCO_3$ as a function of the average $MgCO_3$ crystallite size

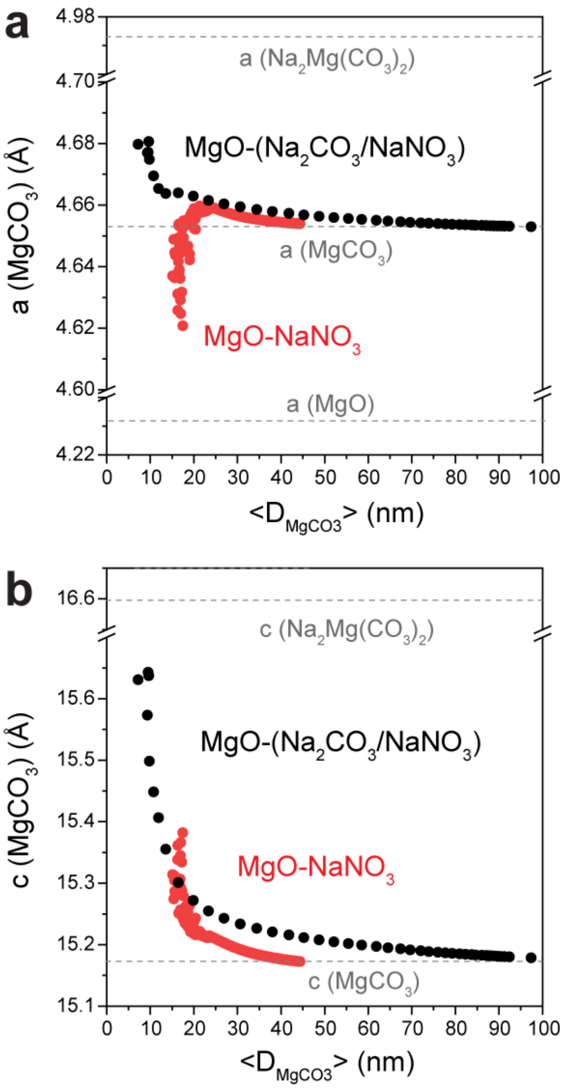

Figure S17. Evolution of the lattice parameters of $MgCO_3$ during the carbonation of MgO-$NaNO_3$ (red dots) and MgO-(Na_2CO_3/$NaNO_3$) (black dots) as a function of the average $MgCO_3$ crystallite size (<D_{MgCO3}>) for (a) the a-parameter and (b) the c-parameter of $MgCO_3$. The respective equilibrium values (value obtained after 60 min of carbonation) of the lattice parameters for MgO, $MgCO_3$ and $Na_2Mg(CO_3)_2$ are included in the plot (grey dashed lines).

Evolution of the lattice parameter of MgO during carbonation

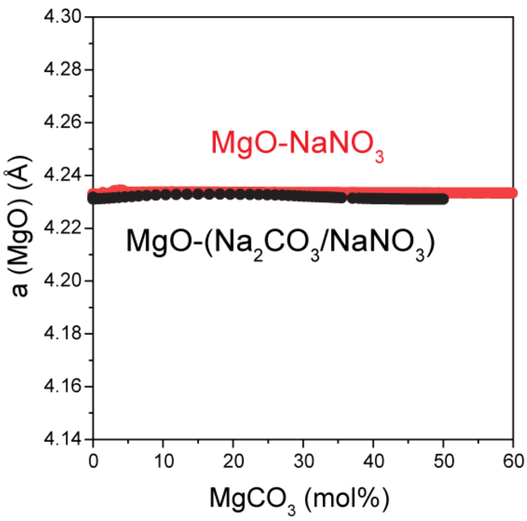

Figure S18. Evolution of the a-parameter of MgO during the carbonation of MgO-(Na_2CO_3/$NaNO_3$) (black dots) and MgO-$NaNO_3$ (red dots). The a(MgO) parameter does not change during the carbonation reaction and is not affected by the presence of Na_2CO_3.

$RbNO_3$ promotion: CO_2 uptake curves obtained in a TGA

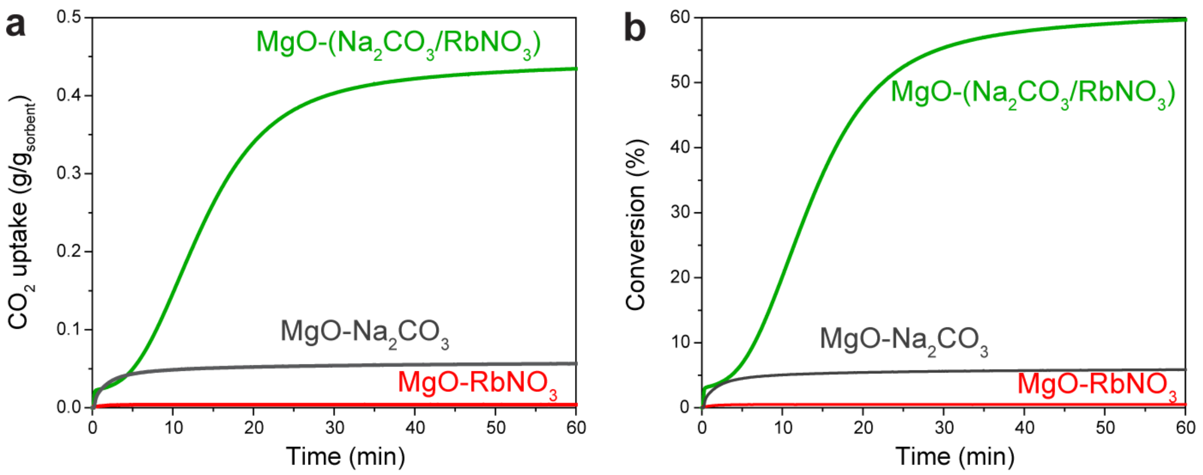

Figure S19. (a) CO_2 uptake and (b) MgO conversion obtained by TGA at 315 °C in CO_2 for MgO promoted with 10 mol% $RbNO_3$ (red), 5 mol% Na_2CO_3 (grey) and a combination of 5 mol% Na_2CO_3 and 10 mol% $RbNO_3$ (green).

Selected XRD patterns collected during the carbonation of (Na_2CO_3/$RbNO_3$)-promoted MgO

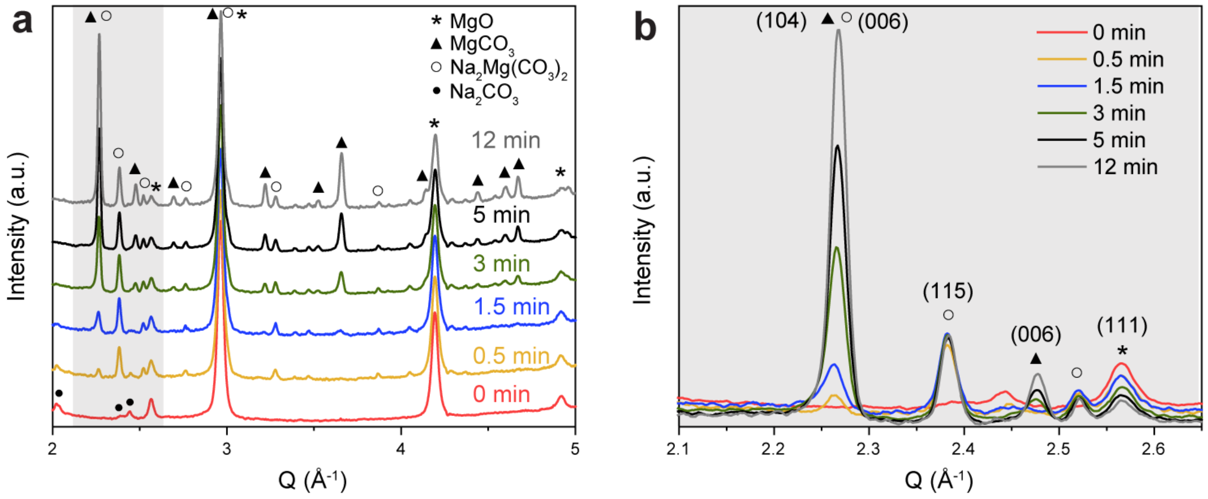

Figure S20. (a) Stacked XRD patterns at various points in time during the carbonation of MgO-(Na_2CO_3/$RbNO_3$); (b) zoom into the gray shaded area in (a).

Selected Rietveld refinements for (Na_2CO_3/$RbNO_3$)-promoted MgO

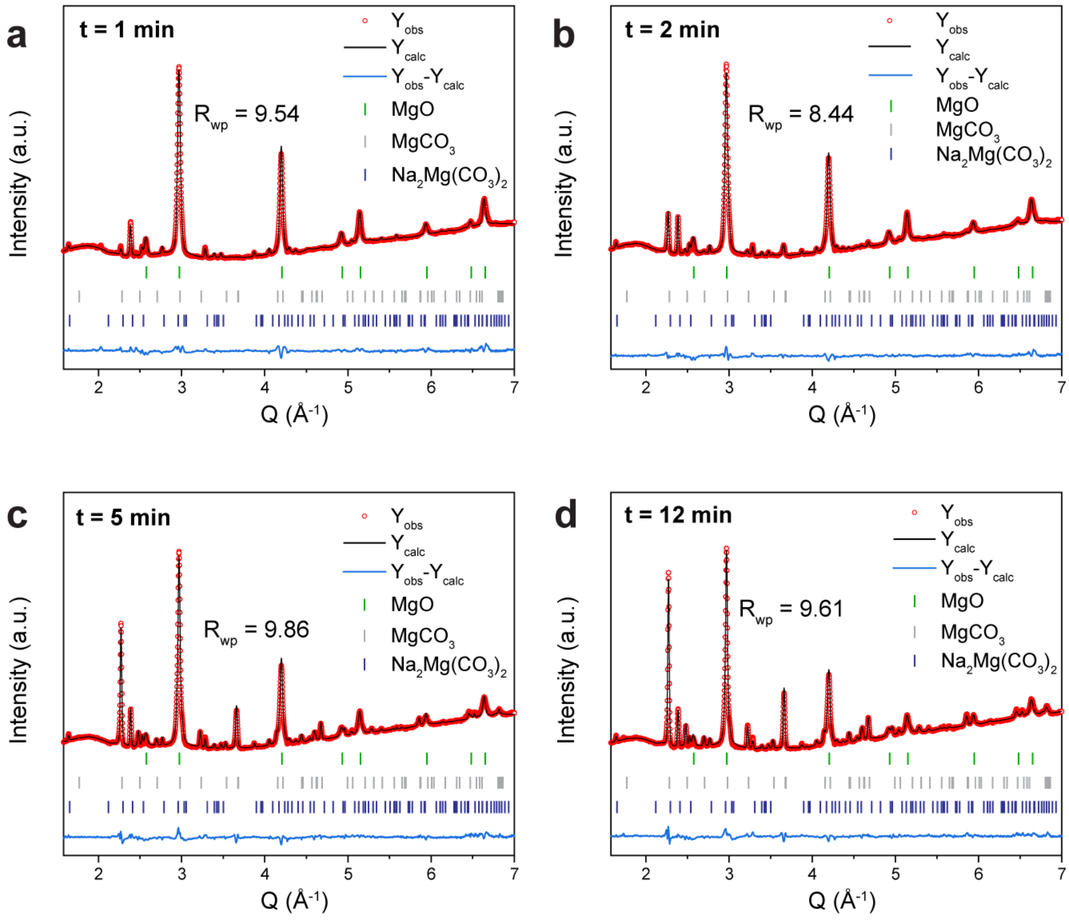

Figure S21. Selected Rietveld refinements for MgO-(Na_2CO_3/$RbNO_3$) at different points in time during carbonation: (a) t = 1 min, (b) t = 2 min, (c) t= 5 min and (d) t = 12 min. Rietveld refinements were performed on selected XRD patterns collected at t = 1, 2, 3, 5, 7.5, 10 and 12 min. The higher background to signal ratio in this sample was due to the fluoresence of Rb at the used wavelength (λ = 0.69668 Å).[3] Points during the first minute of carbonation in Figure 3b, corresponding to $Na_2Mg(CO_3)_2$ formation, were obtained as follows: 1. The relative amount of $Na_2Mg(CO_3)_2$ was determined by integrating the area under the (115) peak of $Na_2Mg(CO_3)_2$. 2. The absolute amount of double salt was calculated by multiplying the relative amount by the absolute amount determined from Rietveld refinement of the XRD pattern at t = 1 min.

In situ XRD data during the carbonation of MgO-Na_2CO_3

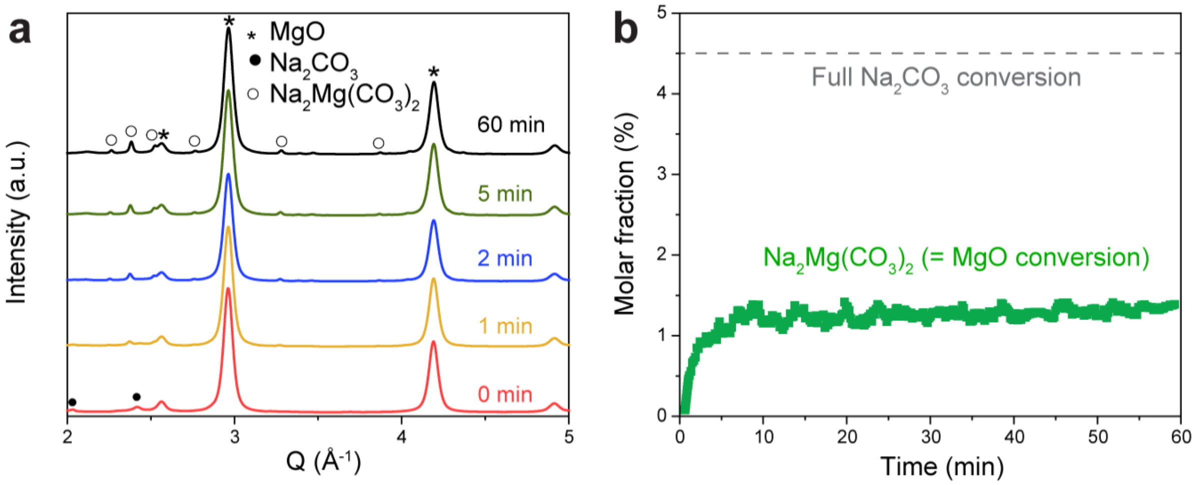

Figure S22. *In situ* XRD data collected during the carbonation of Na_2CO_3-promoted MgO (no molten nitrate) at 315 °C in CO_2. (a) Stacked XRD patterns at various points in time. (b) the molar fraction of $Na_2Mg(CO_3)_2$ (which equals the MgO conversion as no $MgCO_3$ is formed) as a function of time. Note that by the end of the reaction only 1.5 mol% crystalline $Na_2Mg(CO_3)_2$ can be detected (full conversion would correspond to 4.5 mol% $Na_2Mg(CO_3)_2$). No crystalline Na_2CO_3 can be detected after 2 min, implying that the remaining Na_2CO_3 must be amorphous (unclear whether in the form of Na_2CO_3 or $Na_2Mg(CO_3)_2$). Phase quantification was performed as follows: First, the relative amount of $Na_2Mg(CO_3)_2$ was estimated by integrating the area under the (115) peak. The absolute amount of the double salt was then determined by multiplying the relative amount by the absolute amount as determined from Rietveld refinement on the XRD pattern at t = 30 min.

Calculation of the dissolution energy of $MgCO_3$ in $RbNO_3$

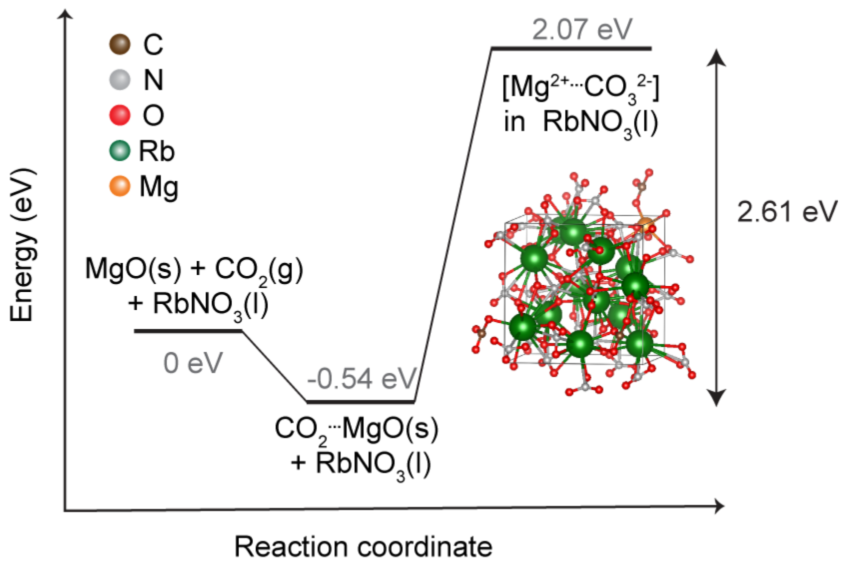

Figure S23. The reaction pathway used to calculate the dissolution energy of $[Mg^{2+}\cdots CO_3^{2-}]$ ion pairs in molten $RbNO_3$: (1) CO_2 adsorption on the MgO(100) surface and (2) dissolution of the adsorbed CO_2 into the $RbNO_3$ melt with formation of a $[Mg^{2+}\cdots CO_3^{2-}]$ ion pair. The graphic represents the structural model used for the AIMD calculation and was visualized using VESTA.[4]

The *in situ* XRD setup

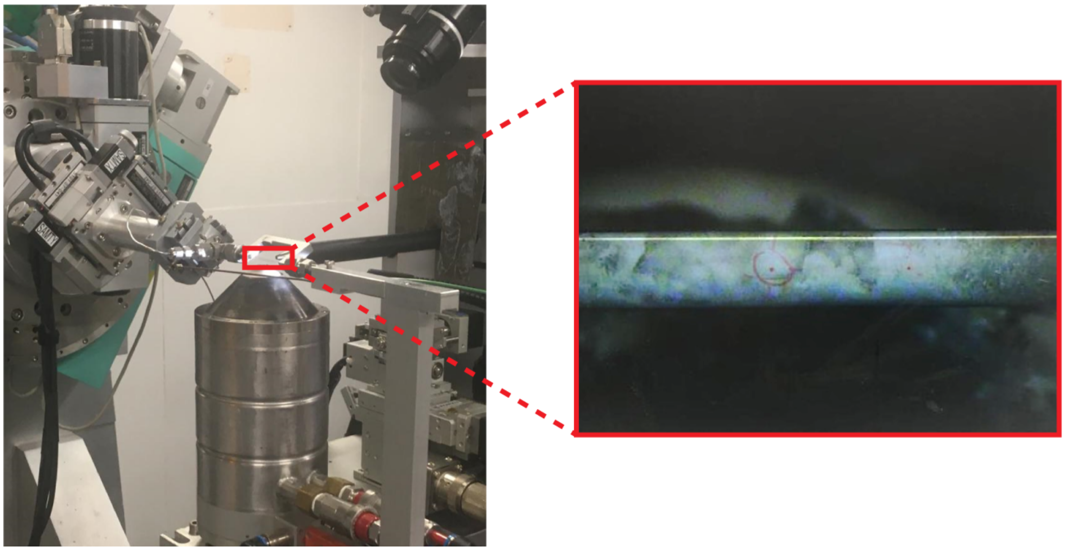

Figure S24. A photograph of the setup used for the *in situ* XRD measurements. The inset shows the sample in the quartz capillary fixed between the quartz wool.

References

(1) Kierzkowska, A. M.; Pacciani, R.; Müller, C. R. CaO-Based CO_2 Sorbents: From Fundamentals to the Development of New, Highly Effective Materials. *ChemSusChem* **2013**, *6* (7), 1130–1148.

(2) Cussler, E. L. *Diffusion: Mass Transfer in Fluid Systems, 2nd Edition*; 1997.

(3) Von Dreele, R. B.; Suchomel, M. R.; Toby, B. H. *Compute X-ray Absorption - Argonne National Laboratory*. https://11bm.xray.aps.anl.gov/absorb/absorb.php (accessed 2024-05-13).

(4) Momma, K.; Izumi, F. *VESTA 3* for Three-Dimensional Visualization of Crystal, Volumetric and Morphology Data. *J. Appl. Crystallogr.* **2011**, *44* (6), 1272–1276.
